# Spectral Characteristics Related to Chemical Substructures and Structures Indicative of Organic Precursors from Fulvic Acids in Sediments by NMR and HPLC-ESI-MS

**DOI:** 10.3390/molecules26134051

**Published:** 2021-07-02

**Authors:** Verónica Gisela López-Martínez, Jorge A. Guerrero-Álvarez, José Gustavo Ronderos-Lara, Mario Alfonso Murillo-Tovar, Jorge Ernesto Solá-Pérez, Ismael León-Rivera, Hugo Saldarriaga-Noreña

**Affiliations:** 1 Centro de Investigaciones Químicas, Instituto de Investigación en Ciencias Básicas y Aplicadas, Universidad Autónoma del Estado de Morelos, Av. Universidad 1001, Cuernavaca C.P. 62209, Morelos, Mexico; girl_akane@hotmail.com (V.G.L.-M.); jguerrero@uaem.mx (J.A.G.-Á.); ronderos92@gmail.com (J.G.R.-L.); ismaelr@uaem.mx (I.L.-R.); 2 CONACYT-Centro de Investigaciones Químicas-IICBA, Universidad Autónoma del Estado de Morelos, Av. Universidad 1001, Cuernavaca C.P. 62290, Morelos, Mexico; mario.murillo@uaem.mx; 3 Facultad de Ingeniería Química, Universidad Autónoma de Yucatán, Periférico Norte Kilómetro 33.5, Tablaje Catastral 13615, Chuburna de Hidalgo Inn, Merida C.P. 97203, Yucatán, Mexico; jsolaperez@yahoo.com

**Keywords:** sediments, organic precursors, fulvic acids, NMR, HPLC-ESI-MS

## Abstract

The aim of this work was to determine Fulvic Acids (FAs) in sediments to better know their composition at the molecular level and to propose substructures and structures of organic precursors. The sediment samples were obtained from a priority area for the conservation of ecosystems and biodiversity in Mexico. FAs were extracted and purified using modifications to the International Humic Substances Society method. The characterization was carried out by 1D and 2D nuclear magnetic resonance (NMR) and high-performance liquid chromatography-electrospray ionization-mass spectrometry (HPLC-ESI-MS) in positive (ESI^+^) and negative (ESI^−^) modes. Twelve substructures were proposed by the COSY and HSQC experiments, correlating with compounds likely belonging to lignin derivatives obtained from soils as previously reported. The analysis of spectra obtained by HPLC-ESI-MS indicated likely presence of compounds chemically similar to that of the substructures elucidated by NMR. FAs studied are mainly constituted by carboxylic acids, hydroxyl, esters, vinyls, aliphatics, substituted aromatic rings, and amines, presenting structures related to organic precursors, such as lignin derivatives and polysaccharides.

## 1. Introduction

The natural organic matter (NOM) present in sediments, soil, and water is considered a very complex chemical matrix, constituted by organic compounds from animal and/or plant origin. NOM is divided into non-humic substances (NHS) and humic substances (HS). The HS are classified into fulvic acids (FAs), humic acids (HAs), and humins (HUs), which are mainly constituted by carbon, hydrogen, and oxygen. Likewise, FAs and HAs also include heteroatoms in their structure, such as nitrogen, sulfur, and phosphorus [1,2,3,4].

In natural and polluted environmental compartments, the physicochemical properties of FAs are of special interest, since they are soluble in the aqueous medium at any pH range and could form hydrogen bonds with water and undergo transfer from the sediment to the aqueous medium. The acidic behavior of FAs is provided by a variety of functional groups such as COOH in fatty and aromatic acids, or substituents from amino acids and phenolic OH groups that come from tannin and lignin structures [5,6,7,8].

The characterization of the structures of humic compounds can provide important information about the primary material and the probable formation mechanism. It represents the starting point in evaluating the capacity for interaction with heavy metals [9,10], pesticides [11,12,13], and different classes of organic compounds, such as polycyclic aromatic hydrocarbons (PAHs) [14,15].

The separation of FAs into fractions with different chemical properties to reduce their heterogeneity remains a challenge. At present, various techniques to isolate FAs have been used, including resin techniques XAD-8 resin and diethyl-aminoethyl cellulose, suggested by the International Humic Substances Society (IHSS), which has a large adsorption capacity, and the adsorbed HS are partly recovered by eluting them with basic solutions [16], chromatographic techniques (such as reversed-phase liquid chromatography and high-performance size exclusion chromatography), ultrafiltration, and electrophoresis [17].

Nuclear Magnetic Resonance (NMR) has been widely used in the characterization of the structural units in different types of lignin preparations and HS. The NMR method is non-destructive and provides direct information about the chemical nature of structural components. However, for HS studies, one-dimensional (1D) and two-dimensional (2D) NMR spectra have low resolution [18,19]. The 1D spectra especially exhibit overlapping signals, which makes structural analysis unreliable and the identification of substructures (combinations of functional group) or structures difficult [5,6,7,20,21,22,23,24,25].

The structural elucidation of HS has been also carried out by mass spectrometry (MS) through electrospray ionization (ESI), either in positive (ESI^+^) or negative (ESI^−^) ion mode, for characterization of NOM. The ESI^−^ mode is more adequate for acid compounds [26]. On the other hand, ESI^+^ mode provides better results for aliphatic and carbohydrate compounds [27]. 

Despite the fact that FAs have an important role in the environment, the molecular composition of these organic compounds in sediments, no studies have yet been conducted in Mexico. Therefore, this work aims to contribute to a better understanding of the chemical structure that constitutes HS in sediment. The analysis involved a process of selective and modified extraction and purification, an improved procedure of conservation of the sample in an inert medium, and a structural characterization by NMR (1D and 2D at 500 MHz) and HPLC-ESI-MS.

## 2. Results and Discussion

### 2.1. Optimization of Conditions for a High Spectral Resolution in NMR

Two samples were collected in November 2018—FABC1 and FABC2. Spectral resolution in NMR experiments was improved through the following: (1) DMSO-*d*_6_ was chosen as the solvent because it prevents the aggregation of FAs components through intermolecular interactions in solution [21,28,29]; (2) the amount of 3 mg in 600 μL used in the preparation of the FABC1 sample contributed considerably to decreasing signals overlap as the mass quantities in a range of 15 to 99.6 mg in DMSO-*d*_6_ showed overlapping of signals or cross peaks in 1D and 2D spectra, respectively [5,6,21,24,25,30]; (3) the increased number of scans in all the experiments compared to that reported in other studies increasing sensitivity [20,21]; (4) the adjustment of acidic to neutral pH in the final eluate decreased selectively the number of signals in the NMR spectra [31].

### 2.2. Identification of Functional Groups through ^1^H NMR

The ^1^H NMR spectrum was used to interpret the chemical characterization of the FAs. The spectrum was grouped from “A” to “F”, according to chemical shifts ranges for HS. Table 1 (Table S1) shows the abundance of each of the groups, predominating the aliphatic region (57%), followed by the intermediate region consisting mainly of methyl and methylene adjacent to carbonyl groups (carboxylic acids, esters) and aromatic rings (19.62%), ethers adjacent to aromatic rings in the lignin structure and polysaccharide rings (14%), and aromatic protons (8.11%). 

### 2.3. Assignment of Substructures through COSY Spectrum NMR

#### 2.3.1. The Aliphatic and Intermediate Regions

**Figure 1:**
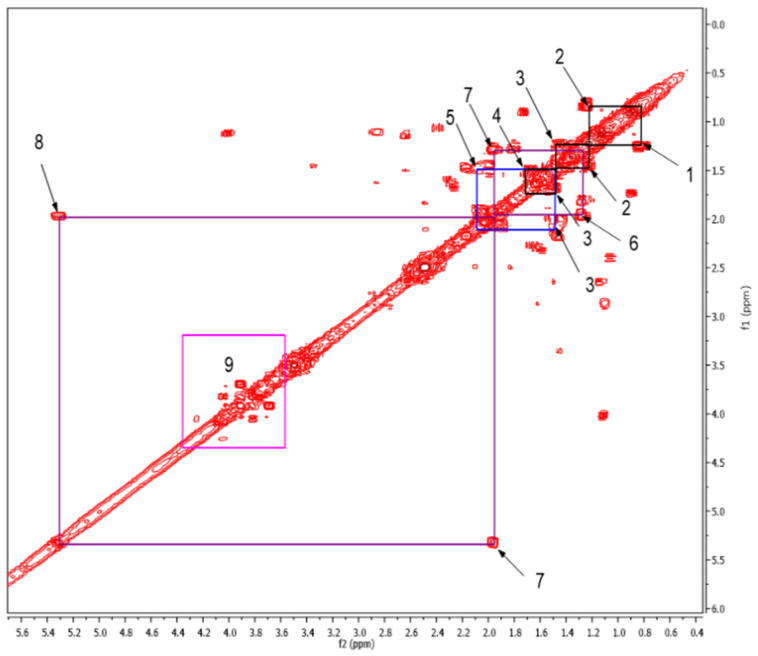
Expansion of the 2D COSY spectrum from 0.4 to 5.6 ppm, which corresponds to the aliphatic and intermediate regions. The box labelled as 9 corresponds to cross peaks of carbohydrates/polysaccharides in positions 2, 3, 4, 5, and 6 (Table S1).

To complement the results obtained with the ^1^H NMR spectra, a Correlation Spectroscopy (COSY) experiment was carried out. The aliphatic and intermediate regions of the COSY spectrum are located between 0.4 and 5.6 ppm (Figure 1). The analysis showed correlations of the aliphatic protons corresponding to cross peaks 1, 2, 3, and 4, and 1, 2, 3, and 5. The couplings between aliphatic protons and vinyl protons correspond to cross peaks 6, 7, and 8. 

From the observed correlations, the substructures A, B, and C were proposed (Table 2). Cross peaks 1–2, 2–3, and 3–4 represented couplings between protons in aliphatic chains. It was proposed that the protons corresponding to 3–4 were attached to a carbonyl group because said correlation is the most displaced towards upfield. Comparing this with correlations 3–5 and 7–8 allowed the proposal of substructure “A”. Meanwhile correlations between cross peaks 1–2, 2–3, and 3–5 suggested that the protons corresponding to correlation 3–5 were attached to a more electronegative functional group than a carbonyl group, which could be an ester, shifting them in the downfield direction, giving rise to substructure “B”. Additionally, cross peaks 6–7 showed correlations between aliphatic protons bound to vinyl protons that corresponded to couplings 7–8, and could result in substructure “C”.

For its part box number 9 corresponds to the carbohydrate/polysaccharide region, which are difficult to identify by NMR, since in this area of the spectrum the signals are overlapped and correlations cannot be established that allow us to know what type of sugars are present in the sample. These results are consistent with that reported by Simpson et al., 2001 [20].

#### 2.3.2. The Aromatic Region

**Figure 2:**
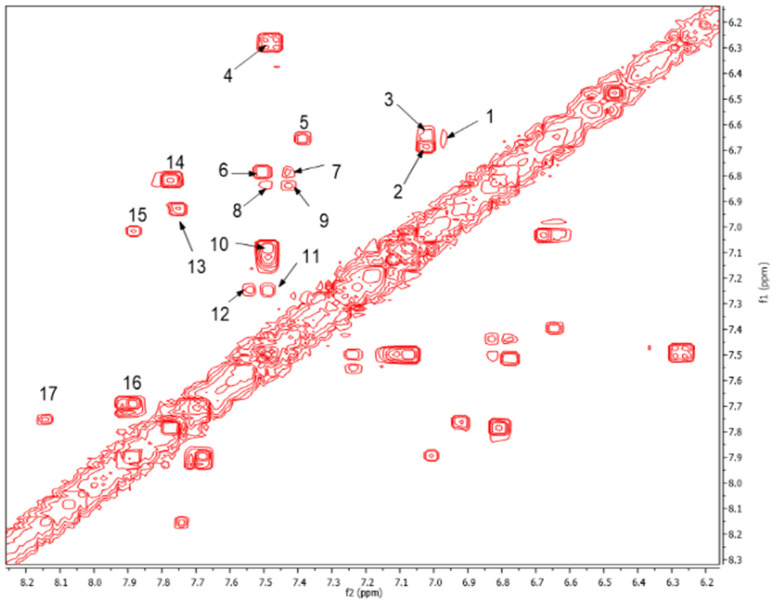
Expansion of the aromatic region of the COSY spectrum from 6.2 to 8.2 ppm. The chemical shifts of the cross peaks numbered from 1 to 17 and their respective couplings are shown in Table 3.

On the other hand, the aromatic region of the COSY spectrum shows 17 sets of cross peaks (Figure 2). Taking into account the chemical shifts of the coupled proton pairs, 12 aromatic substructures were proposed that correlated well with compounds belonging to lignin derivatives obtained from sediments [6,20,32] (Table 3). 

Each group in Table 3 corresponds to aromatic substructures substituted with electro-donating groups adjacent to H_1_ or H_1_* or electro-withdrawing groups adjacent to H_2_ that can be in ortho, meta, or para positions. Group “A” has an aromatic ring substituted with two electro-donating groups in position para, i.e., adjacent to H_1_ and H_1_*, and are the groups OR and R, respectively, where R can be a hydrogen, methoxy, or an aliphatic carbon in a lignin linking group. Consequently, assuming that R is -H or -OCH_3_ group, these could present a mesomeric effect through the unshared pairs of electrons from oxygen, since they are delocalized towards the ring and its electron density increases, especially in the ortho and para positions, shielding to H_1_ and H_1_*. Group “B” shows couplings for vinyl protons where it can be observed that H_2_* is adjacent to the aromatic ring, both for structure “A” as for “B”. Magnetic anisotropy generates protons adjacent or close to aromatic rings that are particularly less shielded due to the magnetic field induced by the electronic currents of these systems. Besides, the induced field is added to that applied through NMR, producing a shift higher than expected. Therefore, H_2_* protons are less shielded than H_1_ and H_2_ protons, showing a difference of more than 1 ppm between them. Group “C” shows di- and tri-substituted aromatic rings, evidencing the highest number of couplings between H_1_ and H_2_ in these organic compounds. These could be adjacent to methoxy or alcohols groups and functional groups such as carboxylic acids, aldehydes, esters, ketones, etc., respectively, showing very similar chemical shifts between them. For group “D” a tetra-substituted aromatic structure is observed with a methoxy group adjacent to H_1_ and an electro-withdrawing group such as carboxylic acids and/or esters groups adjacent to H_2_. Finally, group “E” has di-, tri-, and tetra-substituted benzenes. H_1_ probably is adjacent to -H or -OCH_3_ group and H_2_ could be adjacent to the same groups mentioned for group “D”. However, it is observed that the substitution of the rings with two or three electro-withdrawing groups such as COO-R, displaced the chemical shifts toward downfield in the aromatic region due to a negative inductive effect.

### 2.4. ^13^C Analysis

The *^13^C* functional groups were determined by the following chemical-shift areas [33,34]: alkyl-*^13^C* signals, 0 to 50 ppm; O-alkyl-*^13^C* signals, 50 to 90 ppm; vinyl-*^13^C* signals, 90 to 130 ppm; aromatic-*^13^C* signals, 110 to 165; and carboxyl-*^13^C* signals, 165 to 190 ppm [5,20,30] (Table 4) (Table S2). The signal intensities in the respective chemical shift regions are expressed as a percentage of the total spectra area. The proportion obtained for the *^13^C*-alkyl fraction was 26.5%, O-alkyl 28.5%, vinyl 12.8%, aromatic 19.3%, and carboxyl 12.8% (Figure S1).

The assignment of signals found in the ^13^C spectrum, suggests that alkyl-C and O-alkyl-C are the dominant components, which can probably be used by microorganisms during humification processes [35]. Meanwhile the region of aromatic-*^13^C* includes phenolic-*^13^C*, derived from lignin and tannin and bacterial resynthesized compounds consisting of alkyl-C and carboxyl-C [36,37].

One way to assess the degree of humification of sediments can be done by using the alkyl-C/O-alkyl-C ratio and the aromaticity [38,39]. The alkyl-C/O-alkyl-C ratio calculated in the present study was 0.64, while the aromaticity was 0.22, which indicates that the aliphatic fraction and the one that regulates humification in the studied site. 

### 2.5. Assignment of Functional Groups or Substructures through HSQC Spectrum 

**Figure 3:**
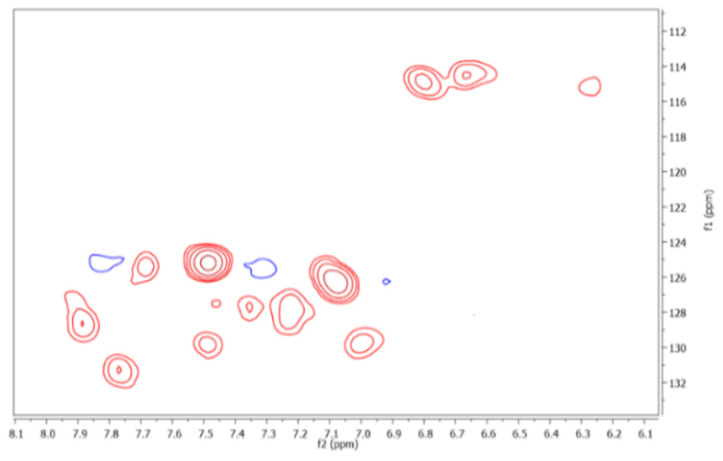
12 cross peaks are observed in the aromatic region of the HSQC spectrum.

Likewise, a 2D HSQC experiment was carried out of the FAs isolated. The results revealed a total of 67 cross peaks. An expanded aromatic region of the HSQC spectrum is shown in Figure 3, observing 12 cross peaks. Thus, according to the chemical shifts established for ^1^H and ^13^C of the FAs [5] and organic compounds [32], the correlations contributed to assigning of functional groups or substructures from organic precursors of the FAs (Table 1, Table 3, and Table 4). 

### 2.6. DOSY Spectrum Analysis

**Figure 4:**
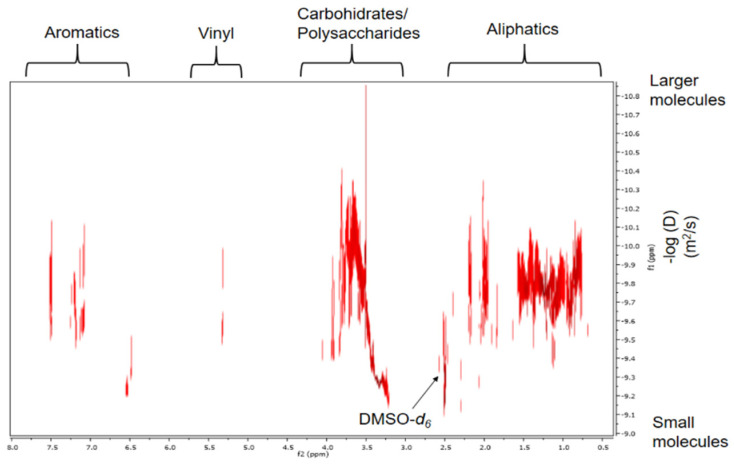
Spectrum corresponding to the DOSY 2D projection.

Finally, the DOSY experiment indicated the incidence that diffusivity has on the separation of a complex mixture, supporting the information that the HS are aggregates of small species and high molecular weight non-macromolecules [21]. The signals corresponding to the components of the polysaccharides presented the slowest diffusion coefficients in solution, which were found in an interval of −log(D) = 9.13–10.33. This means that the structures have larger hydrodynamic radii than the rest of the components in the sample solution and, thus, must be polysaccharides instead of only carbohydrate/saccharide units, which is consistent with the results reported by Simpson et al., 2001 [21]. The main aliphatic components show faster diffusivities (−log(D) = 9.34–10.1) than the polysaccharides and have been identified through the spectra of ^1^H and ^13^C in Table 1 and Table 4, respectively. The fine signal forms for vinyl and lignin derived aromatics with values of −log(D) = 9.46–9.98 and −log(D) = 9.19–10.12, respectively, in FAs suggest that they are relatively low molecular weight, indicating that they are among those with the fastest diffusion coefficients (Figure 4). 

We assume that the apparent molecular weights for carbohydrates are greater than 1500 Daltons (Da), because the diffusion coefficient range is greater for these components than that reported in other studies [21,33]. Furthermore, it should be noted that we obtained a high spectral resolution in the DOSY experiment, which is comparable to the high resolution of the 2D DOSY spectrum obtained in 500 MHz equipment with cryoprobe [21]. This was probably due to the selective and modified extraction and purification process for FAs (Section 3.4 and Section 3.4.1). In addition, to improve the sensitivity of the equipment and increase the spectral resolution, zero filling and forward linear prediction were used. Also, we increased by 14 times the number of scans reported by Simpson et al., 2001 [21].

### 2.7. Assignment of Structures through HPLC-ESI-MS

In order to check the presence of compounds with chemical characteristics similar to those elucidated by NMR, the purified and diluted sediment extract was also analysed by HPLC-ESI-UV and HPLC-ESI-MS. The UV signals indicated chromophore groups in the mixture (Figures S2–S7) (Table 5), since the FAs have functional groups that contain electrons that can present transitions by absorption of specific light. The functional groups responsible for absorption in the ultraviolet region are mainly aromatic rings, which can be substituted in different ways with functional groups, including hydroxyl, carboxylic acids, and aliphatic chains [40]. The 220, 253, and 280 nm wavelengths monitored were chosen according to reported studies [41] (Tables S3 and S4). The absorbances at 280 nm may be indicative of the degree and possible nature of the ring substitution, while the absorption band at 220 and 253 nm has been shown to correlate highly with the degree of aromaticity for HS. Overall, quasi-molecular ions obtained, could be associated with compounds that presented carboxylic acids, aromatic rings, hydroxyls, esters, vinyls, aliphatics, and amines, which is consistent with the groups suggested by the analysis obtained through COSY and HSQC experiments (Table 3). 

On the other hand, some structures with a neutral charge were searched from the database of the NIST (National Institute of Standards and Technology) which were able to relate to the M.W. of the molecular ions corresponding to the quasi-molecular ions with the highest percentage of relative abundance in the mass spectra (Figures S8–S19). This was done in order to propose structures related to possible organic precursors from FAs. Particularly, the analysis of mass spectra obtained by HPLC-ESI (+)-MS at a wavelength of 220 nm, indicated the presence of the compound with molecular formula (C_6_H_13_N, *m*/*z* = 99.4), which probably corresponds to 1,1-dimethylamine-1-butene, and can be protonated (*m*/*z* = 100.4) (Table 5). Similarly, it was found (C_6_H_11_N_3_, *m*/*z* = 125.3), which probably corresponds to 1-(3-aminopropyl) imidazole, p*K*_a_ = 6.5 [41], thus nitrogen of imidazole ring could be protonated corresponding with *m*/*z* = 126.3 [42]. It should be noted that some molecular ions could be related only to one structure in the NIST database. For example, A** and B ** (*m*/*z* = 99.4 and *m*/*z* = 125.3) in Table 5. However, this does not rule out the possibility that some m/z values corresponding to quasi-molecular ions can be observed at different retention times (t_R_), suggesting the presence of homologous organic series in the mixture of FAs.

Meanwhile C, D, and E compounds have a molecular formula of C_x_H_y_O_z_ and could be ionized in positive mode because these have oxygen atoms rich in electrons [43] (Table 5). In comparison with NMR experiments, the proposed structure of 1,2-Benzenedicarboxylic acid, diundecyl ester for D, which has a molecular weight of 474 g/mol (Table 5), likely correspond with a substructure elucidate from the cross peaks at 7.70, 7.92, 7.75, and 8.14 ppm for ^1^H and 131.28 and 128.64 ppm for ^13^C. These results are consistent with functional groups reported by a model of the hypothetical path of fragmentation obtained from Suwannee River Fulvic Acid (SRFA) [8], which was derived using electrospray ionization/ion trap multistage tandem mass spectrometry (ESI/MST/MS). In this model, water losses are characteristics of the dehydration of alcohols and the formation of anhydrides. CO_2_ losses corresponding to decarboxylation and CO losses from esters were also observed. Such a model points to lignin precursors because the final ionic product at 109 *m*/*z* was O-dihydroxybenzene and this is indicative of lignin structure [22]. Also, the quasi-molecular ion at 365.1 *m*/*z* was obtained by negative ion mode from the sediment sample. The proposed formula for its molecular ion (366.1 *m*/*z*) was C_17_H_18_O_9_ (Table 5), which is congruent with the structure suggested by Witt et al., 2009, that showed an ion quasi-molecular weight of 365.088 *m/z* was obtained from a sample of FAs extracted from dissolved organic matter (DOM) [44]. 

The α-hydrogens in carbonyl components and hydrogen bound to oxygen are acids, which are easily unprotonables in ESI^−^ mode [27]. Although carbohydrates are preferentially protonated and efficiently ionized in the positive ion mode, the presence of polysaccharides in sediments could be evidenced through 1893.6 m/z obtained by HPLC-ESI(−)-MS, and it has been reported that neutral oligosaccharides can be identified by negative electrospray ionization collision-induced dissociation tandem mass spectrometry (ESI-CID-MS/MS) for the analysis of their sequence with various bonds, linear and branched structures, and with different residues of saccharides [45]. Compounds related to 1894.6 g/mol and 113.7 g/mol, corresponding to J and K, respectively, were not found.

## 3. Experimental

### 3.1. Materials and Methods

For sampling, a 25 cm long auger with 2 cm diameter was used, samples were stored in amber glass containers with airtight lids. The samples were processed in an electric grinder and a 2.00 mm sieve was used to sift them. For the extraction and purification process, columns with 2 cm diameter were used. The reagents such as sodium hydroxide (NaOH), hydrochloric acid (HCl), and hydrofluoric acid (HF) were analytical grade and the resins were Supelite^TM^ XAD-8 and DOWEX^®^ 50W-X8 (hydrogen form, 50-100 mesh, Merck, Gernsheim, Germany). The extracts were filtered using fine-pore filter paper (Science Med, Aldrich, Mexico City, Mexico). For the NMR experiments, anhydrous hexadeuterated dimethylsulfoxide (DMSO-*d*_6_, Aldrich, Mexico) and a J. Young valve NMR tube with a diameter of 5 mm (Wilmad, for 500 MHz, S.P. Scientific, Stone Ridge, NY, USA) were used. For the HPLC-ESI-MS experiments, H_2_O and acetonitrile grade HPLC were used.

### 3.2. Description of the Site

The sampling site is located within PEUBC which is a priority area for the conservation of ecosystems and biodiversity in the city of Cuernavaca, Morelos, Mexico. This park is crossed by a stream of water, whose tributary comes from the recharges of the underground aquifers of the Chichinautzin Biological Corridor. The study site was selected for the following reasons: (1) this place does not present deforestation, which contributes to the existence of degraded organic compounds that are derived from plants such as Amate prieto (*Ficus continifolia*), Yellow amate (*Ficus petiolaris*), Fresno (*Fraxinus uhdei*), Clavellino (*Pseudobombaxellipticum*), Pochote (*Ceiba aesculifolia*), and Ahuehuete (*Taxodium mucronatum*), which is 250 years old [48]; (2) PEUBC has high values of carbon fixation, which reveal the conversion of inorganic carbon into organic carbon by living organisms, representing an autochthonous source of organic matter [49]; (3) presents an edaphology type Feozem or Phaeozem, thus, it is found normally under a process of accumulation of humus [50]; and (4) has two types of geology; the first is Basalt (*Igneous rock*), which is the most abundant extrusive rock formed from molten magma, i.e., fine grained and dark coloured rocks, containing 50% feldspar and 50% ferromagnesian minerals (pyroxens and olivine), and the second is sandstone *(Sedimentary*) which are sedimentary rocks formed from sediments showing different stages of formation: weathering, transportation, deposition or sedimentation, and diagenesis [51]. 

### 3.3. Sampling 

Two samples were collected on 16 November 2018 (FABC1) and (FABC2) and 1 kg of sediment was collected in each sampling. The sediment core was taken at depths of 10–20 cm using an auger. The samples were immediately stored and transferred to the laboratory in amber glass containers with airtight lids at 25 °C. Subsequently, the samples were dried at room temperature inside the extraction hood for 72 h.

### 3.4. Fulvic Acids Extraction 

Both FABC1 and FABC2 samples were treated in the same way. Initially they were dried at room temperature. For each sample the HS were extracted following the procedure established by IHSS [52], with slight modification. In brief, the sediment sample was crushed in a mortar. Subsequently, to obtain homogeneous particle size, the sample was processed in an electric grinder. Then, the sample was passed through a sieve and stored in an amber container with a tight lid at room temperature. After 24 h, 150 g of sample was placed in an Erlenmeyer flask and deionized water was added to provide a ratio of 1 mL of liquid/0.5 g of dry sample. Later, keeping a constant stirring, the pH was adjusted to 2 by the addition of 1 M HCl.

The solution was then equilibrated with 0.1 M HCl, until a ratio of 10 mL/1 g of dry sample was obtained. After 1 h of stirring, the solution was allowed to stand and after 14 h, supernatant was removed and labelled as “FA1 extract”; later it was stored at 4 °C. This was done with the purpose of extracting FAs, since they are soluble in acidic and basic medium, while HAs are only soluble in basic medium. On the other hand, HUs are not soluble in basic medium or in acid medium. After the extraction of FA1, the sediment that remained in the Erlenmeyer flask was neutralized by the addition of 1M NaOH. Subsequently, 0.1 M NaOH was added under a nitrogen (N_2_) atmosphere to avoid oxidation of HS during extraction [53], resulting in a final ratio of extractant to sediment of 10:1, obtaining a suspension. This suspension was stirred for 4 h and then allowed to settle. After 16 h, the supernatant was collected and acidified with 6 M HCl to a pH of 1, to decant the HAs. The suspension was allowed to settle until two well-defined phases were observed (after 16 h). The supernatant was collected and labelled as “FA2 extract”. Finally, the FA1 and FA2 extracts were centrifuged at 1000 rpm and passed through filter paper. Thus, both extracts were accumulated and this mix was labelled as FA3.

#### 3.4.1. Fulvic Acids Purification 

FA3 was passed through a XAD-8 resin, previously conditioned with 0.1 M NaOH and deionized water, and packed in a column, according to the proposed methodology for Thurman and Malcolm 1981 [52]. These conditions allowed the retention of FAs through hydrophobic interactions. Later, the resin was washed with 16 mL of deionized water to remove some remaining salts. Finally, retained FAs were eluted with 24 mL of 1 M NaOH and 72 mL of deionized water, and immediately acidified to a pH of 1 with 6 M HCl. After acidification, HF was added to obtain a final concentration of 0.3 M HF. The HCl-HF treatment successfully reduced the ash contents of the HS without modifying their chemical composition and structure significantly [35]. Also, the HF treatment has been proposed for removing paramagnetic Fe salts from HS; these paramagnetic Fe salts are the main source of interference in NMR [31]. The solution HCl-HF that contained the FAs was extracted again as FA3. The eluate solution was then passed through 24 mL of the H^+^ saturated cation exchange resin and subsequently 24 mL of 0.3 M NaOH was added for the extraction of FAs in their protonated form, since the resin has sulfonic acid as an active group in styrene-divinylbenzene, which easily donates its protons. The eluate was lyophilized in a BenchTop Pro-Sp Scientific kit for 24 h at −80 °C and 50mTorr. To give a plus to the extraction and purification method established by the IHSS, the solute was dissolved in a volume of 47 mL of deionized H_2_O and 3 mL of 0.1 M NaOH to adjust the pH from acidic to neutral in the solution. This was done in order to specifically analyze the aromatic rings of the FAs that contain hydroxyl groups in their structure, since according to potentiometry studies at a pH of 6.4, the FAs present a formation of 50% of these species [53]. Subsequently, the sample was lyophilized again. 

### 3.5. Instrumental Techniques 

#### 3.5.1. Nuclear Magnetic Resonance (NMR)

1D and 2D NMR spectra were recorded on a Bruker Avance III-500 MHz spectrometer, Billerica, MA, USA) of a sample containing 3 mg of FABC1 dissolved in 600 µL of DMSO-*d*_6_, using a J. Young valve NMR tube within an Argon atmosphere, to avoid degradation of the sample. As a reference for ^1^H and ^13^C assignments, DMSO signals were used (2.5 and 39.5 ppm, respectively). For structural analysis, the experiments ^1^H, ^13^C, COSY, and ^1^H-^13^C HSQC were acquired according to the methodology established by Simpson et al., 2001 [20]. DOSY pulse sequence was LED with bipolar gradient pulse pair, two spoil gradients were acquired, according to the methodology established by Simpson et al., 2007 [21]. In order to improve the spectral resolution in all experiments, the number of scans was increased compared to those previously reported in the literature [20,21]. Additional details for all experiments are shown in Table 6.

With the intention of preserving the sample, after each NRM experiment the FABC1 sample was refrigerated at a temperature between −10 to −30 °C in an Ar atmosphere. Subsequently, it was placed at room temperature for 24 h before carrying out each of the NMR experiments.

#### 3.5.2. High-Performance Liquid Chromatography-Electrospray Ionization-Mass Spectrometry (HPLC-ESI-MS)

FABC2 sample was used for chromatographic analysis. A liquid chromatography (Agilent Technologies 1260) coupled to mass spectrometry (Agilent 6120 Infinity Technologies was used. The HPLC-ESI-MS analyses were based on Stenson 2008 [54] and Frauendorf and Herzschuh 1998 [55]. Briefly, chromatographic separation was performed on a C_18_ waters column (250 mm × 4.6 mm, 5 µm) using an isocratic elution with a mobile phase composed of H_2_O: ACN (50:50) at a flow rate of 0.3 mL·min^−1^ with a sample injection of 5 µL. In order to increase the ionization efficiency, 0.1% ammonium formate was added prior to negative mode electrospray (pH = 8). For positive ion mode, 0.1% formic acid was used (pH < 6). Chromatograms were acquired at 220, 253, and 280 nm. Then a mass spectrometric analysis was carried out, for each wavelength, with a quadrupole at SCAN mode acquisition (100−2000 *m*/*z*). Both positive (FABC2-1) and negative (FABC2-2) electro spray ionization were applied to 1 mg of dissolved extract at water. Quasi-molecular ions M^+^H for ESI^+^ and M^−^H for ESI^−^ were monitored to obtain fragment spectra. The nebulization gas flow rate was set at 12 mL·min^−1^, the ion spray was set at 3000 V, and the temperature was kept at 350 °C. 

## 4. Conclusions

This work is the first characterization of Fulvic Acids in Mexico through NMR and HPLC-ESI-MS from the PEUBC, a priority area for the conservation of ecosystems and biodiversity. The improvements in the methodology (extraction, purification, and characterization) method proposed by the IHSS for the analysis of these HS was modified reducing signal overlapping and improving signal-to-noise ratio. The improved spectral resolution obtained in the DOSY spectrum obtained in 500 MHz cryoprobe equipment was probably due to the selective and modified extraction and purification processes.

The results obtained in the different NMR experiments allowed us to acquire structural information of the fulvic acids present in the studied area. The humification process in the sediment studied suggests the highest contribution of the alkyl-C fraction.

## Figures and Tables

**Table 1:** Specific ranges for signals in ^1^H NMR spectrum corresponding to the functional groups that constitute fulvic acids. As well as the comparison of the integration percentages obtained from sections “A” to “F” in said spectrum.

Hydrogen Type	δ ppm	Sections
Primary alkyl, secondary alkyl, methyl in position β to alcohol, β methylene adjacent to olefines, aliphatic protons on β and γ to aromatic rings.	0.64–1.64	A (57%)
Methyl and methylene adjacent to carboxylic acids, methyl and methylene adjacent to carbonyls, esters, aromatic rings, olefins.	1.64–3.0	B (19.62%)
Ethers adjacent to aromatic rings in the lignin structure, CH at positions 2, 3, 4, and 5 and CH_2_ at position 6 in carbohydrate/polysaccharide rings.	3.5–4.31	C (14%)
Hydroxyl groups adjacent to aromatics rings.	4.31–4.64	D (0.194%)
Vinyls.	5.27–5.80	E (0.99%)
Aromatic protons.	6.15–8.19	F (8.11%)

**Table 2:** Suggested substituents according to the correlations found between aliphatic protons and vinyl protons.

A	B	C
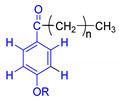	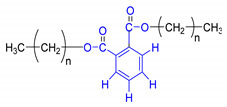	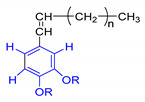

n = undetermined number of methylene groups; R = H, methoxy or an aliphatic carbon in a lignin linking group.

**Table 3:** Suggested aromatic substructures of the FAs corresponding to lignin derivatives, which were obtained by COSY and HSQC experiments.

Group A				
No.	^1^H_1_ (ppm)	^1^H_1_* (ppm)	^13^C (ppm)	Structure *
1 2 3	6.66 6.69 6.65	6.97 7.02 7.01	114.53, 125.40, 129.81, 126.30	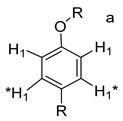
**Group B**				
**No.**	**^1^** **H_1_ or ^1^H_2_ (ppm)**	**^1^** H_2_* **(ppm)**	**^13^** **C** **(ppm)**	Structure *
4	6.30	7.50	115.19, 129.81, 125.19, 127.49, 114.81	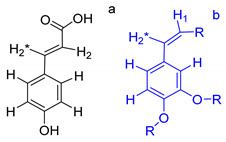
**Group C**				
**No.**	**^1^H_1_ (ppm)**	**^1^H_2_** **(ppm)**	**^13^C** **(ppm)**	Structure *
5 6 7 8 9 10 13 14 15	6.65 6.77 6.79 6.81 6.81 7.09 6.93 6.82 7.02	7.39 7.52 7.43 7.49 7.43 7.49 7.75 7.78 7.88	129.81, 125.19, 127.49, 114.81, 125.40, 126.30, 131.28, 128.64	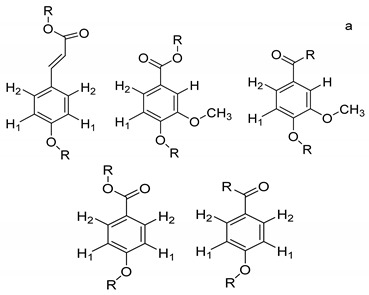
**Group D**				
**No.**	**^1^** **H_1_ (ppm)**	**^1^** **H_2_** **(ppm)**	**^13^** **C** **(ppm)**	Structure *
11 12	7.25 7.25	7.49 7.51	127.85, 127.70, 129.19, 127.49, 114.81	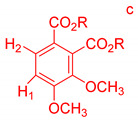
**Group E**				
**No.**	**^1^** **H_1_ (ppm)**	**^1^** **H_2_** **(ppm)**	**^13^** **C** **(ppm)**	Structure *
16 17	7.70 7.75	7.92 8.14	128.64, 131.28	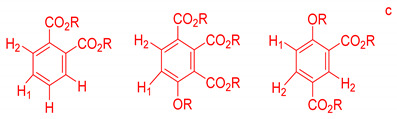

* R = H, methoxy or an aliphatic carbon in a lignin linking group; H_1_ and H_1_* are adjacent to electro-donating groups, H_2_ is adjacent to electro-withdrawing groups and H_2_* is adjacent to the aromatic rings [6] ^a^, [20] ^b^, [32] ^c.^.

**Table 4:** Specific ranges for signals in *^13^C* corresponding to the functional groups that constitute fulvic acids.

Carbon Type	δ
	ppm
Aliphatic *^13^C* adjacent to carbonyls, Aliphatic *^13^C* adjacent to aromatic carbons, and Aliphatic *^13^C* adjacent to olefins	0–50
Ether *^13^C* adjacent to aromatic rings in the lignin structure	50–90
CH at positions 2, 3, 4, and 5 and CH_2_ at position 6 in carbohydrate/polysaccharide rings	57–76
Vinyl *^13^C*	90–130
Phenolic *^13^C*	110–165
Carbonyl *^13^C*	165–190

**Table 5:** Suggested molecular formulas from the spectra obtained by HPLC-ESI-MS.

ESI (+)			
λ = 220 nm			
Quasi-Molecular and Molecular Ion	Molecular formula	t_R_ (min)	Absorbance
	C_6_H_13_N	5.025	***
A * = 100.4 *m*/*z*	M.W. = 99.1741 g/mol	9.028	***
A ** = 99.4 (a)		53.136	***
	C_6_H_11_N_3_	5.025	***
B * = 126.3 *m*/*z*	M.W. = 125.1716 g/mol	9.028	***
B ** = 125.3 (a)		53.136	***
	C_7_H_6_O_2_	6.073	***
C * = 123.2 *m*/*z*	M.W. = 122.1213 g/mol		
C ** = 122.2 (a)			
	C_30_H_50_O_4_	13.492	***
D * = 475.3 *m*/*z*	M.W. = 474.7156 g/mol		
D ** = 474.3 (a)			
	C_9_H_12_O_2_	14.965	***
E * = 149 *m*/*z*	M.W. = 148.1586 g/mol		
E ** = 148.0 (a)			
**ESI (−)**			
**λ = 220 nm**			
**Quasi-Molecular and Molecular Ion**	**Molecular formula**	**t_R_ (min)**	**Absorbance**
	C_5_H_6_O_3_	5.794	***
	M.W. = 114.0993 g/mol	6.127	***
	C_6_H_10_O_2_	9.148	***
	M.W. = 114.1424 g/mol	10.095	***
A * = 113.1 *m*/*z*	C_7_H_14_O		
A ** = 114.1 (a)	M.W. = 114.1855 g/mol		
	C_7_H_11_O_5_	6.127	***
	M.W. = 175.1672 g/mol	15.995	***
B * = 174.9 *m*/*z*	C_10_H_7_O_3_		
B ** = 175.9 (a)	M.W. = 175.1687 g/mol		
	C_30_H_48_O_5_	6.127	***
C * = 487.2 *m*/*z*	M.W. = 488.6991 g/mol		
C ** = 488.2 (a)			
		6.127	***
	C_28_H_54_O_11_		
	M.W. = 566.7218 g/mol		
D * = 565.2 *m*/*z*	C_36_H_70_O_4_		
D ** = 566.2 (a)	M.W. = 566.9386 g/mol		
	C_7_H_5_O_3_	9.148	***
E * = 136.9 *m*/*z*	M.W. = 137.1207 g/mol		
E ** = 137.9* (a)			
	C_11_H_10_O_2_	9.148	***
F * = 173.0 *m*/*z*	M.W. = 174.1959 g/mol		
F ** = 174.0 (a)			
	C_11_H_8_O_3_	10.095	***
G * = 187.1 *m*/*z*	M.W. = 188.1794 g/mol		
G ** = 188.1 (a)			
H * = 201.1 *m*/*z*	C_13_H_14_O_2_	10.095	***
H ** = 202.1 (a)	M.W. = 202.2491 g/mol		
I * = 197.1 *m*/*z*	C_10_H_14_O_4_	15.995	***
I ** = 198.1* (a)	M.W. = 198.2158 g/mol		
J * = 1893.6 *m*/*z*		15.995	***
J ** = 1894.6			
	Undetermined		
		29.053	****
K * = 112.7 *m*/*z*	Undetermined		
K ** = 113.7			
	C_17_H_18_O_9_	29.053	****
L * = 365.1 *m*/*z*	M.W. = 366.088 g/mol		
L ** = 366.1 (b)			

[46] ^a^ [47] ^b^, * quasi-molecular ion, where z = 1, ** molecular ion, M.W. = molecular weight, λ: wavelength, t_R_: retention time, *** absorbance observed, and **** absorbance not observed. Only the peaks of the mass spectra corresponding to the wavelength of 220 nm were analyzed.

**Table 6:** Optimized parameters in each of the NMR experiments.

NMR Experiments	Scans	Additional Information
1D ^1^H	3084 vs. 32 [20]	
1D ^13^C	279,352 vs. 16,000 [20]	Line broadening 1 Hz. Pulse delay 2 s.
2D COSY	96 vs. 32 [20]	
2D HSQC	752 vs. 16 [20]	145 Hz one-bonded heteronuclear J coupling
2D DOSY	14,336 vs. 1024 [21]	

## Data Availability

The data presented in this study are available on request from the corresponding author.

## Supplementary Materials

The following are available online, Figure S1: 1D ^13^C-NMR spectrum. The DMSO-*d*_6_ signal appears at 39.5 ppm, Figure S2: Sample FABC2-1, λ = 220 nm, Figure S3: Sample FABC2-2, λ = 220 nm, Figure S4: Sample FABC2-1, λ = 253 nm, Figure S5: Sample FABC2-2, λ = 253 nm, Figure S6: Sample FABC2-1, λ = 280 nm, Figure S7: Sample FABC2-2, λ = 280 nm, Figure S8: Sample FABC2-1, tR = 5.025 min., Figure S9: Sample FABC2-1, tR = 6.073 min., Figure S10: Sample FABC2-1, tR = 9.208 min., Figure S11: Sample FABC2-1, tR = 13.492 min., Figure S12: Sample FABC2-1, tR = 14.965 min., Figure S13: Sample FABC2-1, tR = 53.136 min., Figure S14: Sample FABC2-2, tR = 5.794 min., Figure S15: Sample FABC2-2, tR = 6.127 min., Figure S16: Sample FABC2-2, tR = 9.148 min., Figure S17: Sample FABC2-2, tR = 10.095 min., Figure S18: Sample FABC2-2, tR = 15.995 min., Figure S19: Sample FABC2-2, tR = 29.053 min., Table S1: Specific ranges for signals in 1H corresponding to the functional groups that constitute fulvic acids, integration percentages obtained from the sections “A” to “F” in said spectrum and possible structures, Table S2: Specific ranges for signals in 13C corresponding to the functional groups that constitute fulvic acids, Table S3: Retention times (tR) corresponding to the wavelengths of 220, 253 and 280 nm for ESI+ mode, Table S4: Retention times (tR) corresponding to the wavelengths of 220, 253 and 280 nm for ESI- mode, Table S5: Peaks from A to E obtained from the mass spectra in SCAN mode (Figures S8–S19) using ESI+ mode. The m/z ratios of A to E, their respective retention times (tR) and the percentage of relative abundance of each peak are shown, Table S6: Peaks from A to L obtained from the mass spectra in SCAN mode (Figures S20–S25) using ESI- mode. The m/z ratios of A to L, their respective retention times (tR) and the percentage of relative abundance of each peak are shown.

## Sample Availability

The results obtained in the study are available from the authors.
