# Supplementary material for: Spectral Characteristics Related to Chemical Substructures and Structures Indicative of Organic Precursors from Fulvic Acids in Sediments by NMR and HPLC-ESI-MS
Source: Molecules. 2021 Jul 2;26(13):4051. doi: 10.3390/molecules26134051 (PMC8272027; doi:10.3390/molecules26134051)
Supplement: Supplementary file 1 [file molecules-26-04051-s001.zip › molecules-1189145-supplementary.pdf]

Supplementary material

Table S1 Specific ranges for signals in ^{1}H corresponding to the functional groups that constitute fulvic acids, integration percentages obtained from the sections "A" to "F" in said spectrum and possible structures.

Hidrogen type	functional group	δ ppm	Group
• Primary alkyl.<br>• Secondary alkyl.<br>• Methyl in position β to Alcohol.<br>• β-methylenes adjacent to olefins.<br>• Aliphatic protons on β and γ to aromatic rings.	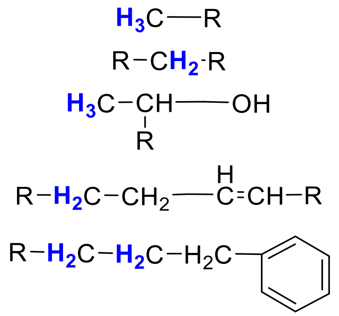	0.64-1.64	**A** 57%
• methyls and methylenes adjacent to carboxylic acids.<br>• methyls and methylenes adjacent to carbonyls.<br>• methyls and methylenes adjacent to esters.<br>• Methyls and methylenes adjacent to aromatics rings.<br>• methyls and methylenes adjacent to olefins.	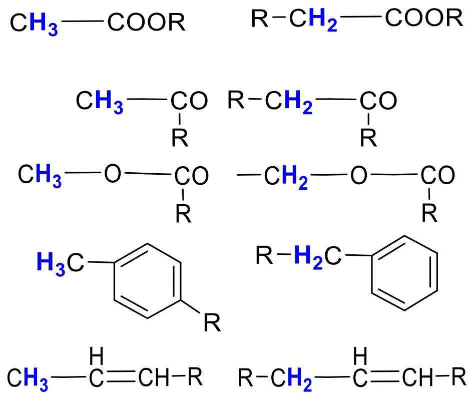	1.64-3.0	**B** 19.62%

• Ethers adjacent to aromatic rings in the lignin structure.<br>• CH at positions 2, 3, 4, and 5 and CH_2 at position 6 in carbohydrate/polysaccharide rings.	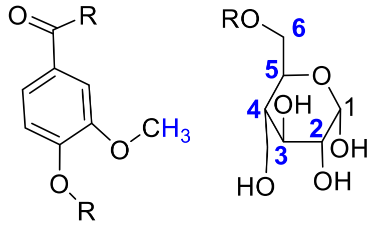 	3.5-<br>4.31	**C**<br>14%
• Hydroxyl groups adjacent to aromatics rings.	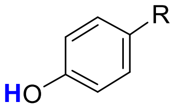 	4.31-<br>4.64	**D**<br>0.194%
• Vinyls.	$R-HC=CH-R$	5.27-<br>5.80	**E**<br>0.99%
• Aromatic protons.	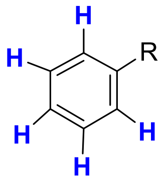 	6.15-<br>8.19	**F**<br>8.11%

Table S2. Specific ranges for signals in ^{13}C corresponding to the functional groups that constitute fulvic acids.

Carbon type	functional group	δ ppm
• Aliphatic C adjacent to carbonyls.	$OC(R)-CH_3$	0-50
• Aliphatic C adjacent to aromatic carbons.	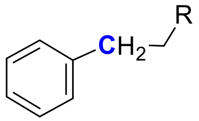	
• Aliphatic C adjacent to olefins	$R-H_2C-HC=CH-CH_2-R$	
• Ether C adjacent to aromatic rings in the lignin structure.	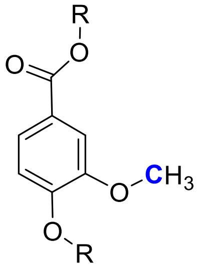	50-90
• CH at positions 2, 3, 4, and 5 and CH_2 at position 6 in carbohydrate/polysaccharide rings	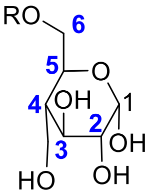	57-76
• Vinyl C	$R-HC=CH-R$	90-130

•	Phenolic C	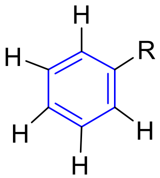	110-165
•	Carbonyl C	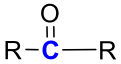	165-190

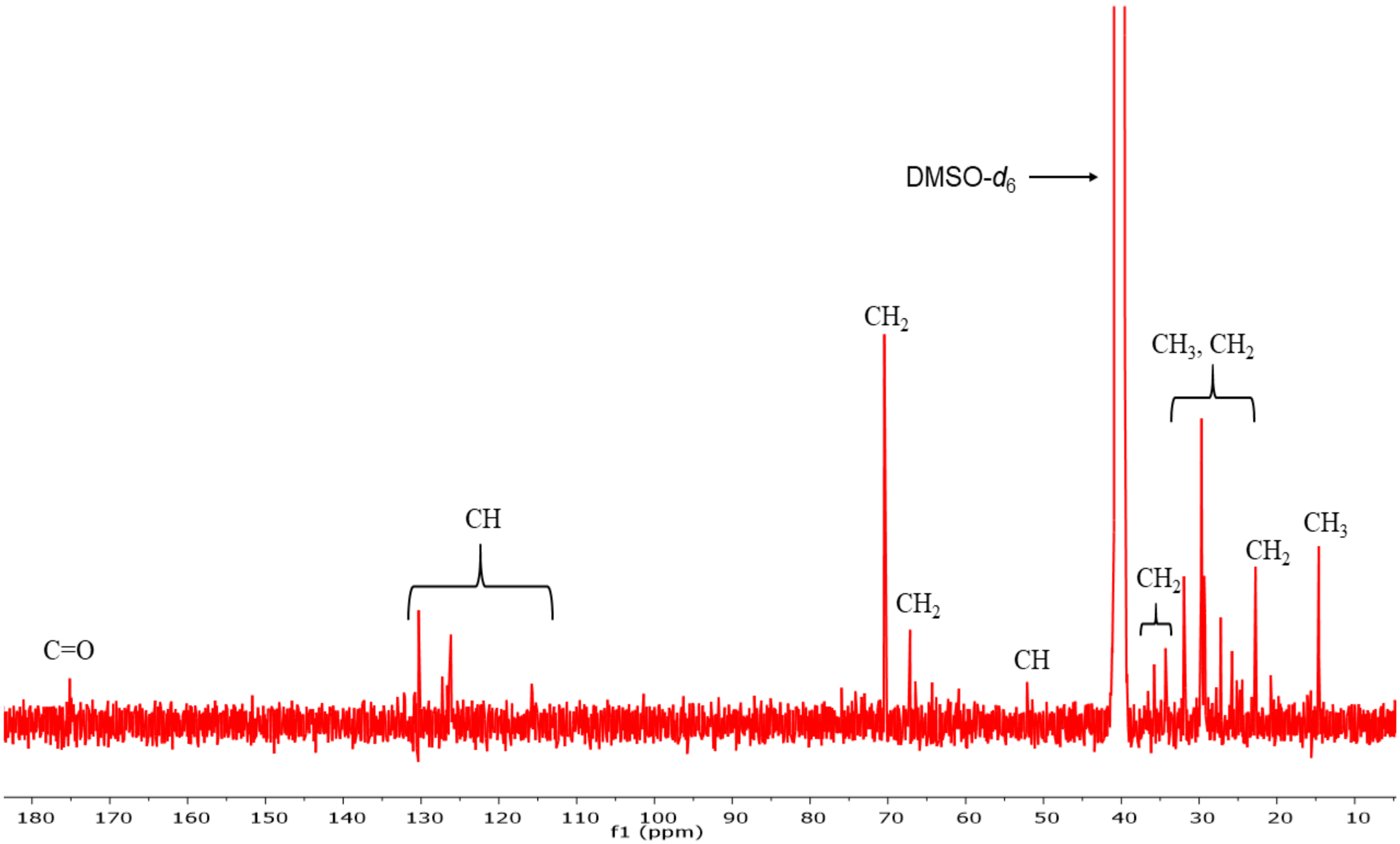

Fig. S1. 1D ^{13}C-NMR spectrum. The DMSO-d_6 signal appears at 39.5 ppm.

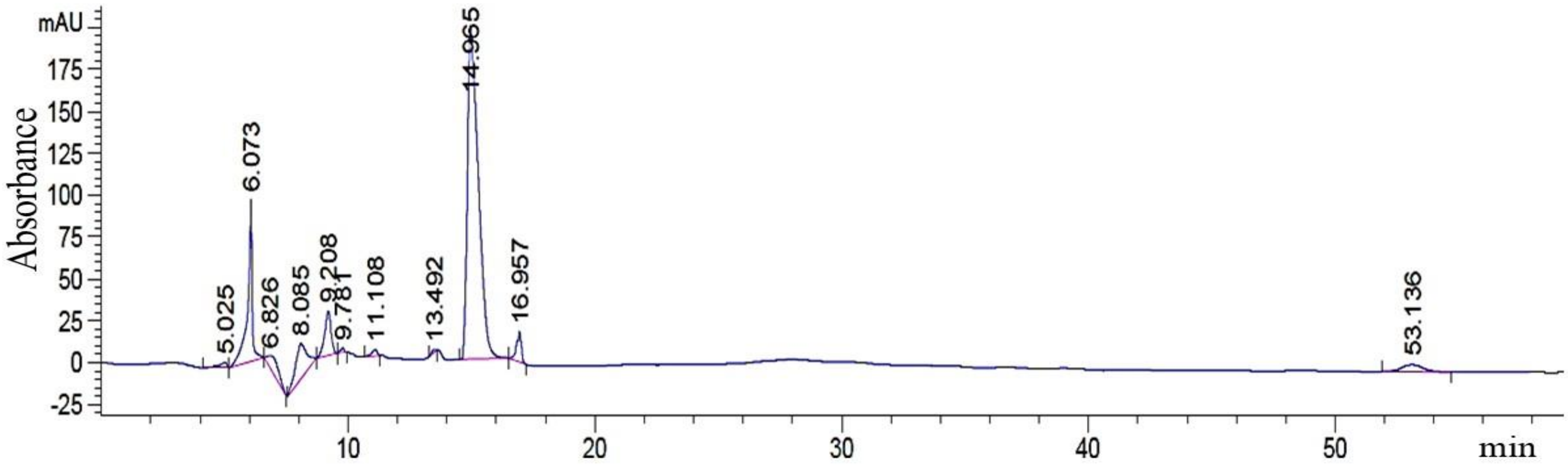

Fig. S2. Sample FABC2-1, λ=220 nm.

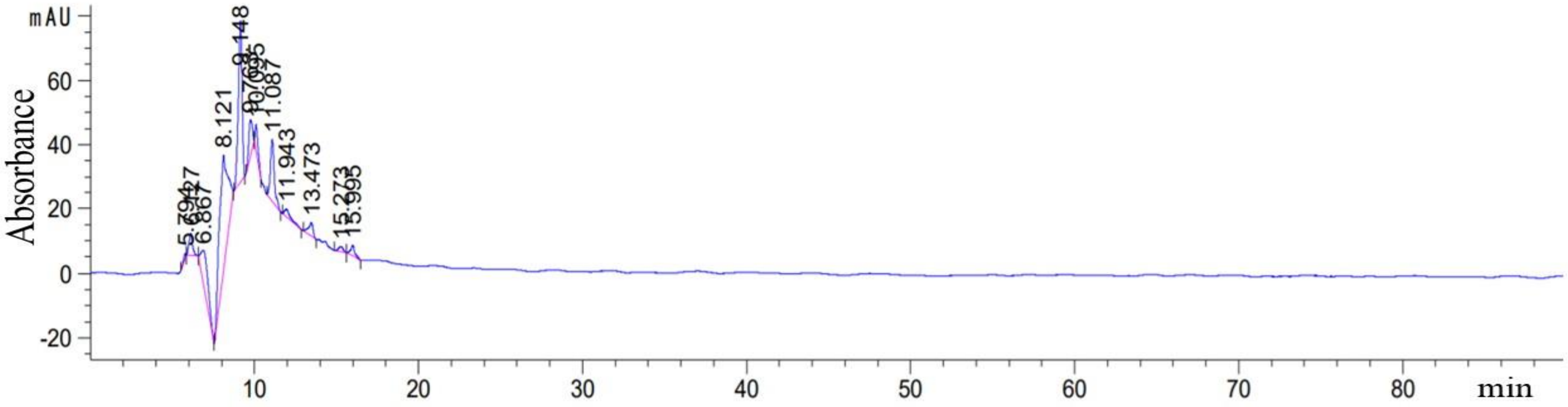

Fig. S3. Sample FABC2-2, λ=220 nm.

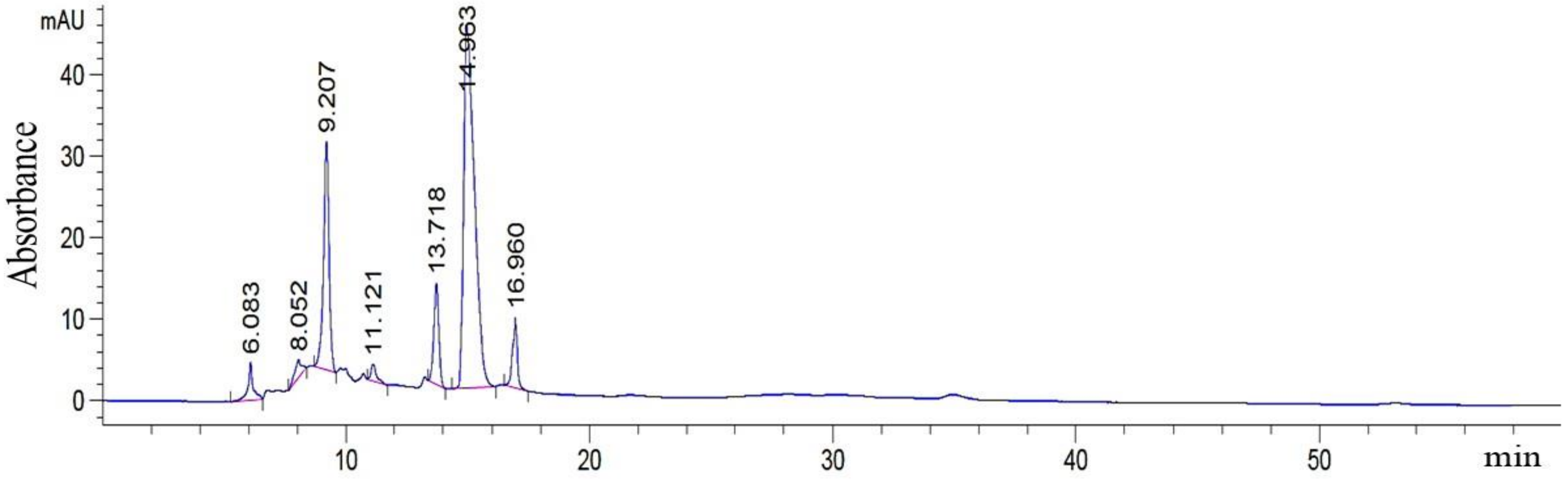

Fig. S4. Sample FABC2-1, λ=253 nm.

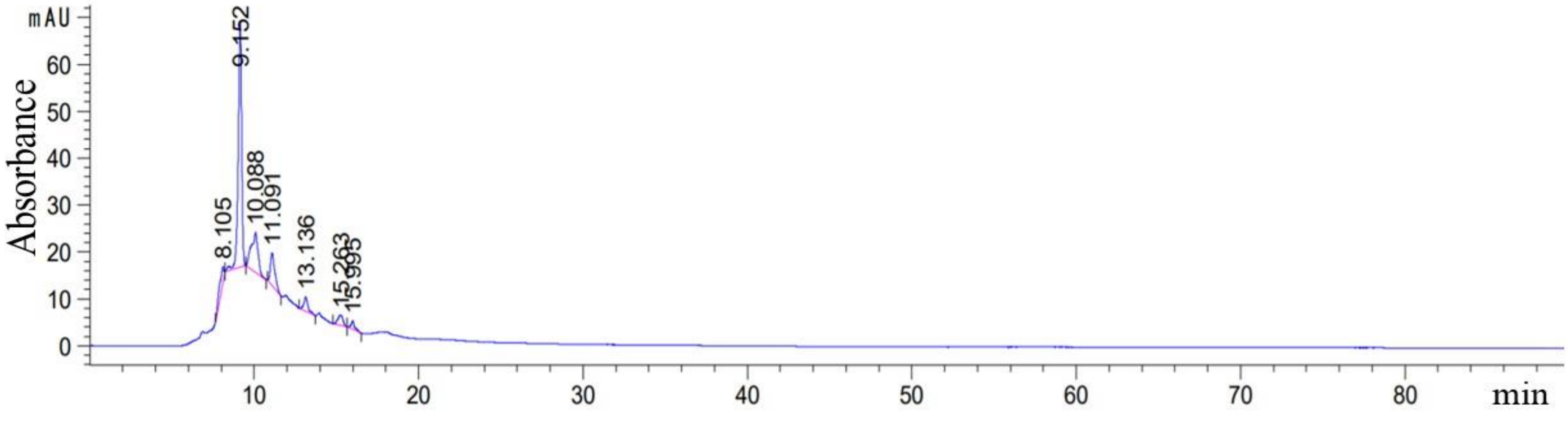

Fig. S5. Sample FABC2-2, λ=253 nm.

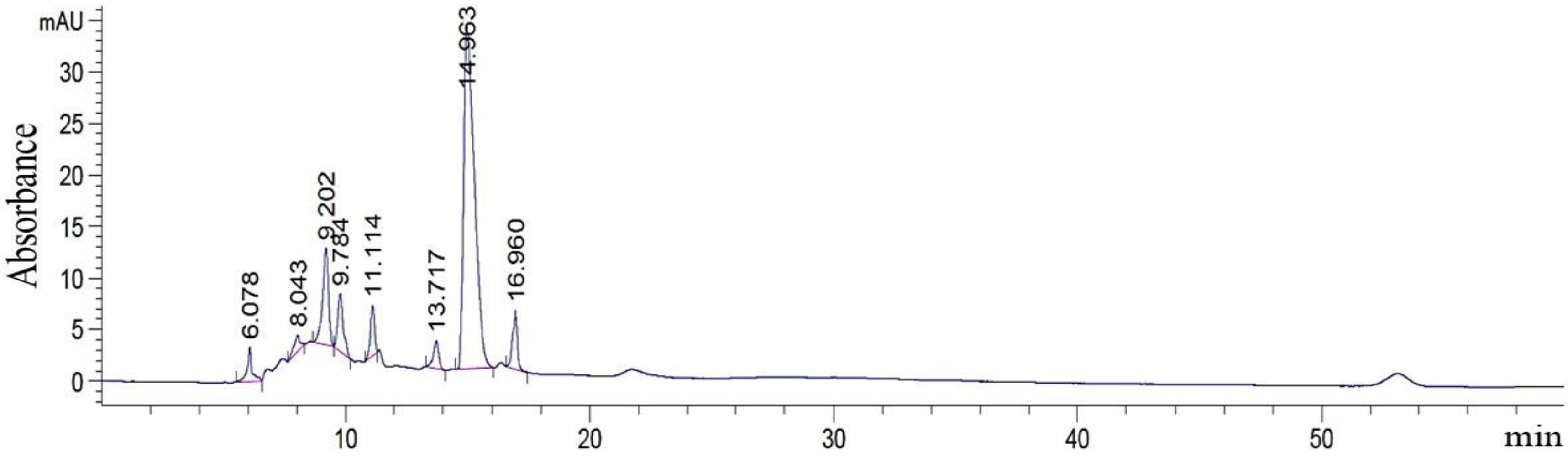

Fig. S6. Sample FABC2-1, λ=280 nm.

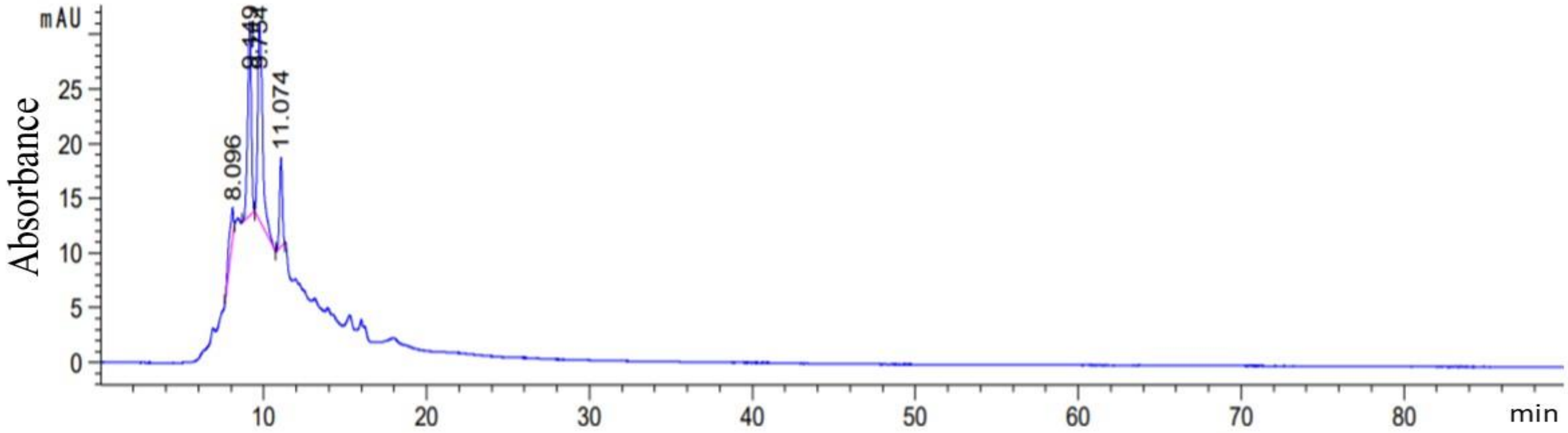

Fig. S7. Sample FABC2-2, λ=280 nm.

Table S3. Retention times (t_R) corresponding to the wavelengths of 220, 253 and 280 nm for ESI+ mode.

λ=220 nm	λ=253 nm	λ=280 nm
t_R (min)	t_R (min)	t_R (min)
*5.025		
*6.073	6.083	6.078
6.826		
8.085	8.052	8.043
*9.208	9.207	9.202
9.781		9.784
11.108	11.121	11.114
*13.492	13.718	13.717
*14.965	14.963	14.963
16.957	16.960	16.960
*53.136		

*Mass spectra were extracted from retention times marked.

Table S4. Retention times (t_R) corresponding to the wavelengths of 220, 253 and 280 nm for ESI- mode.

λ=220 nm	λ=253 nm	λ=280 nm
t_R (min)	t_R (min)	t_R (min)
*5.794		
*6.127		
6.867		
8.121	8.105	8.096
*9.148	9.152	9.149
9.763		9.754
*10.095	10.088	
11.087	11.091	11.074
11.943		
13.473	13.136	
15.273	15.263	
*15.995	15.995	

*Mass spectra were extracted from retention times marked.

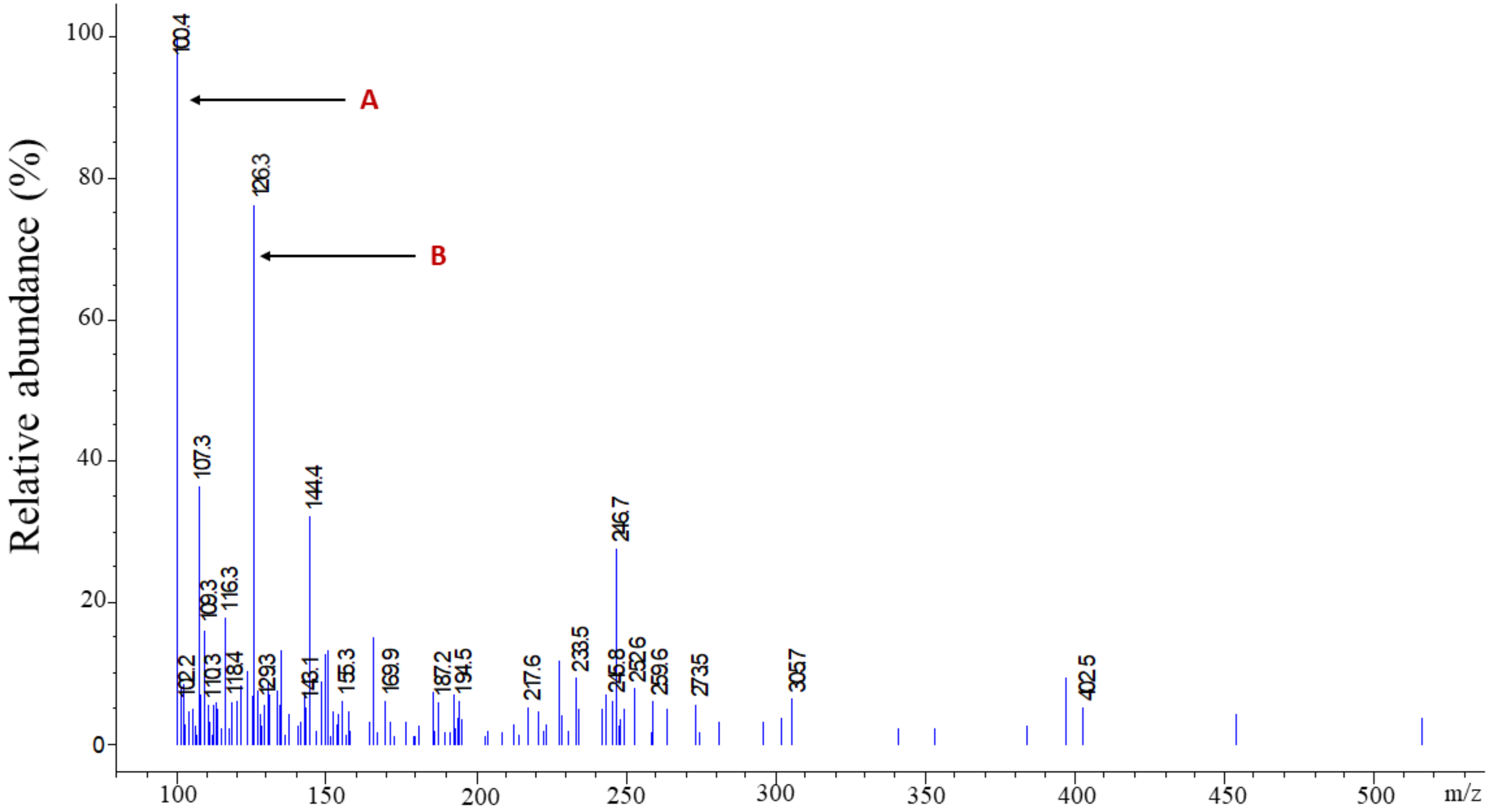

Fig. S8. Sample FABC2-1, t_R=5.025 min.

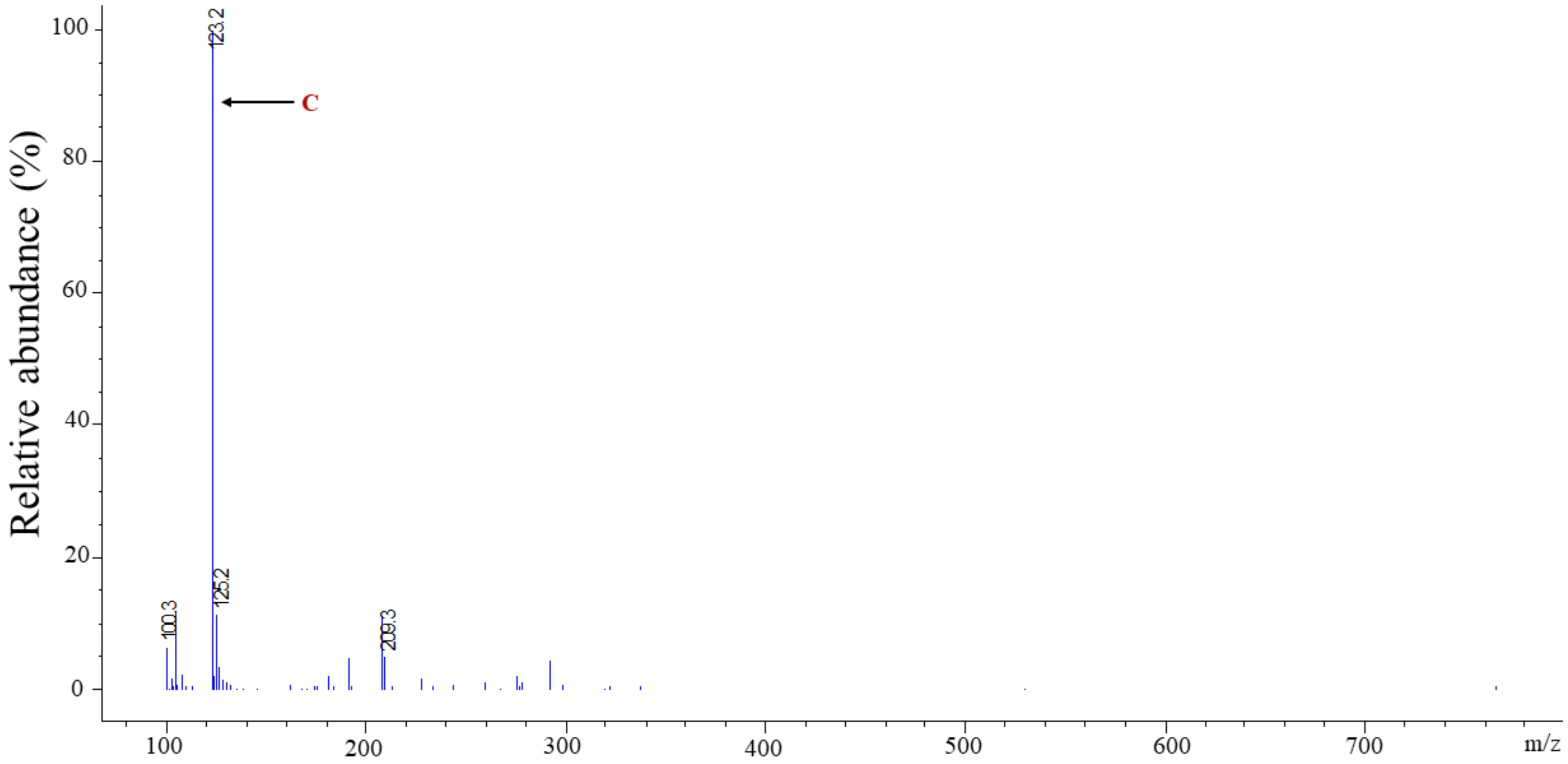

Fig. S9. Sample FABC2-1, t_R=6.073 min.

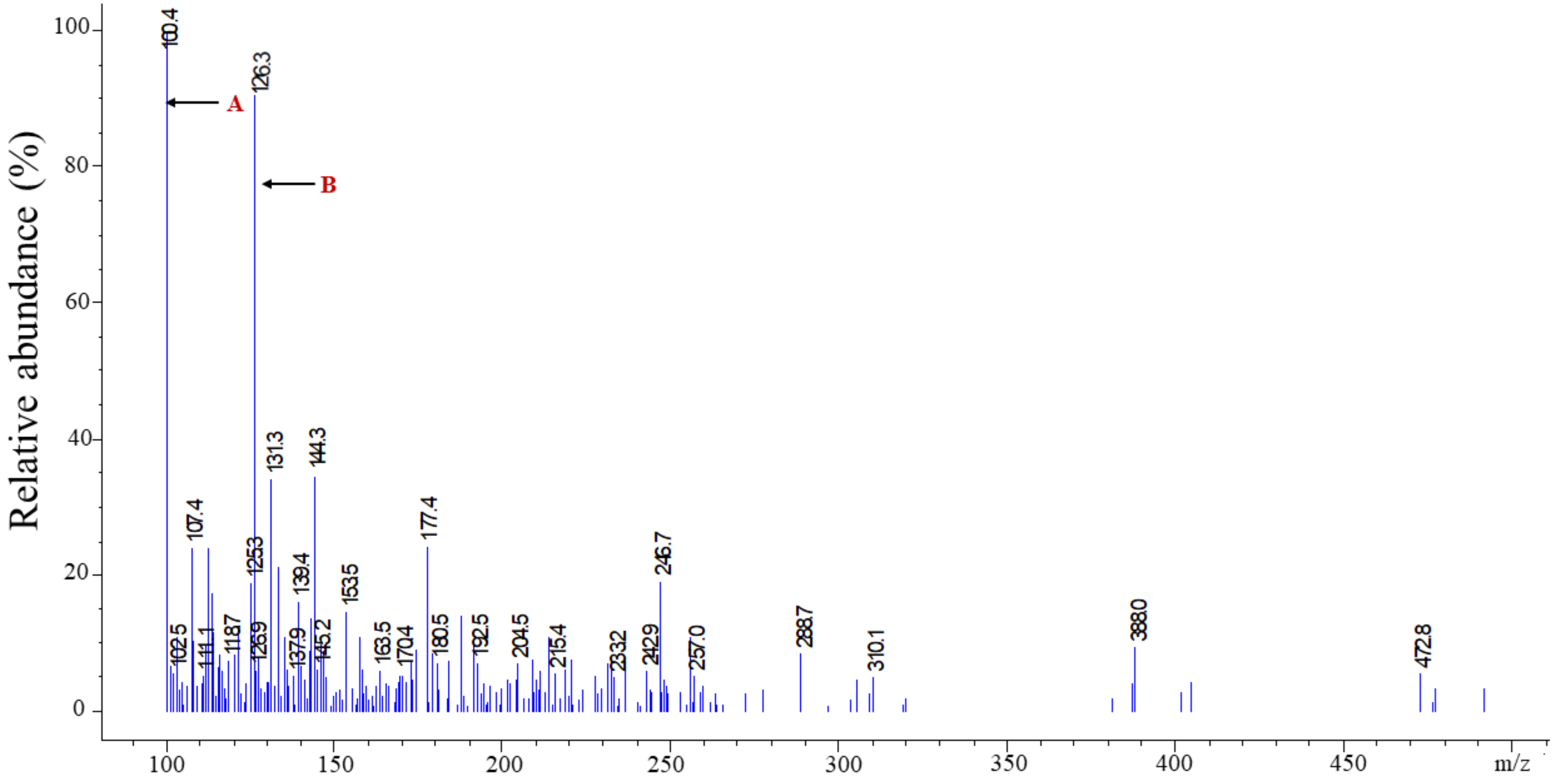

Fig. S10. Sample FABC2-1, t_R=9.208 min.

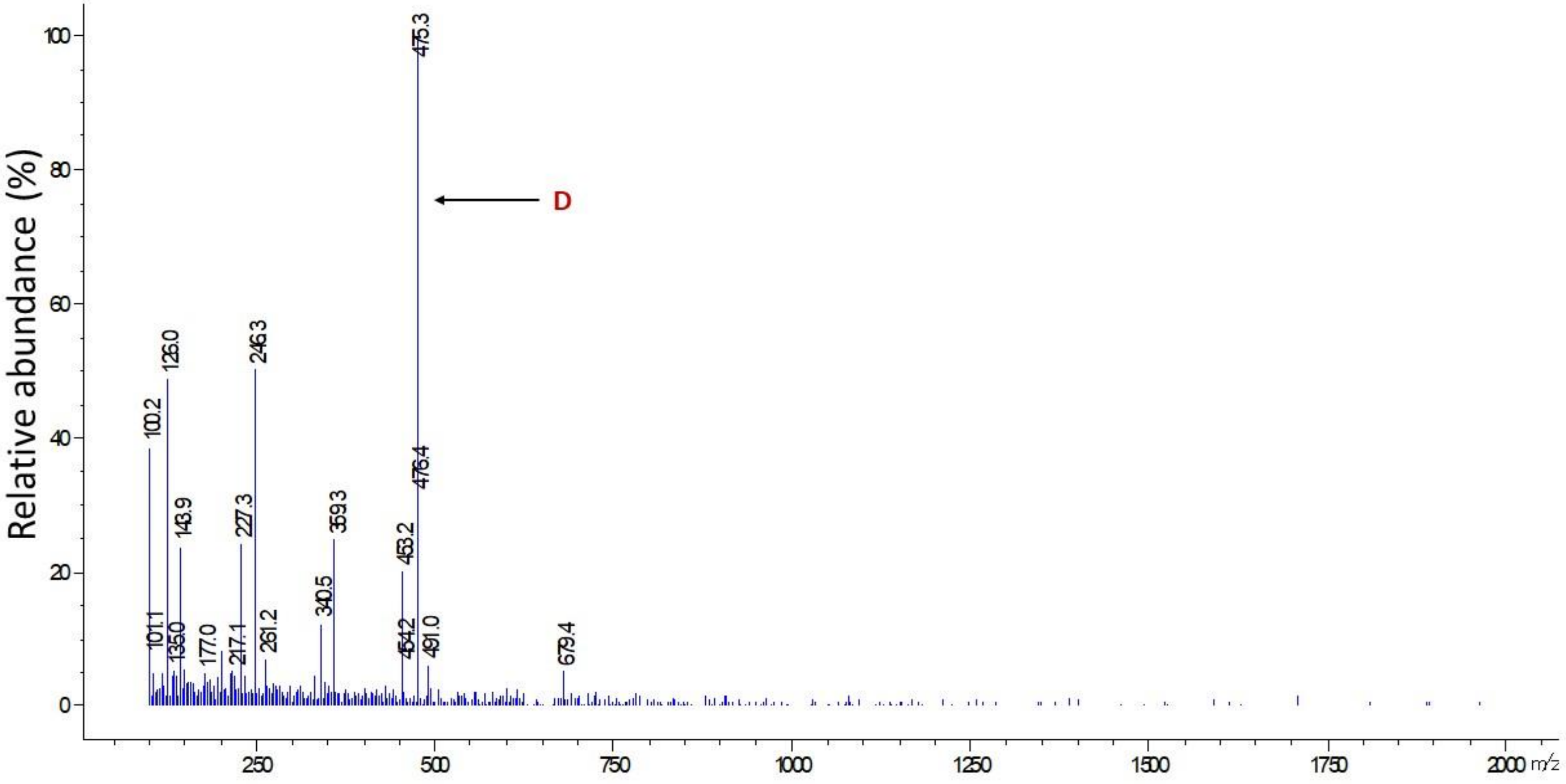

Fig. S11. Sample FABC2-1, t_R=13.492 min.

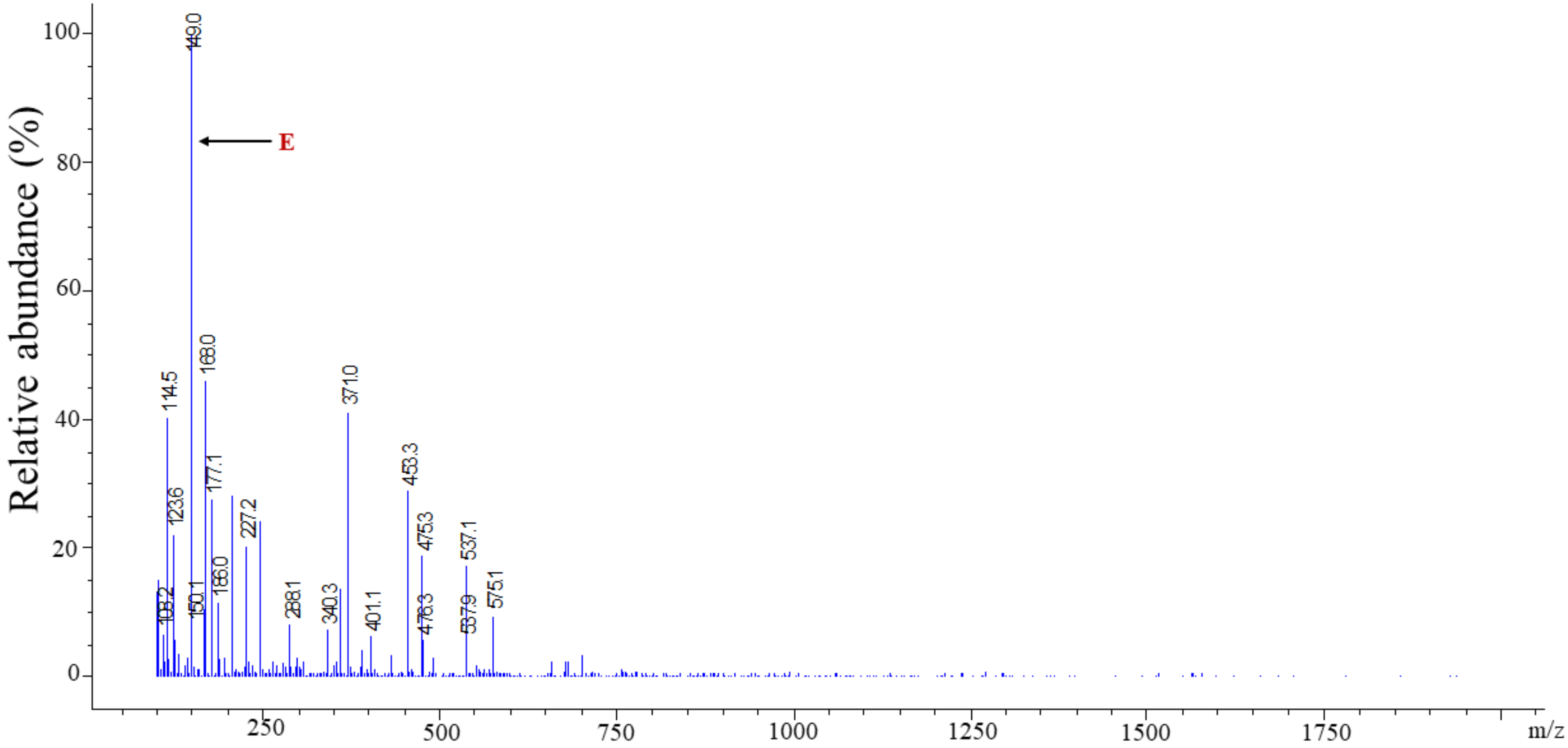

Fig. S12. Sample FABC2-1, t_R=14.965 min.

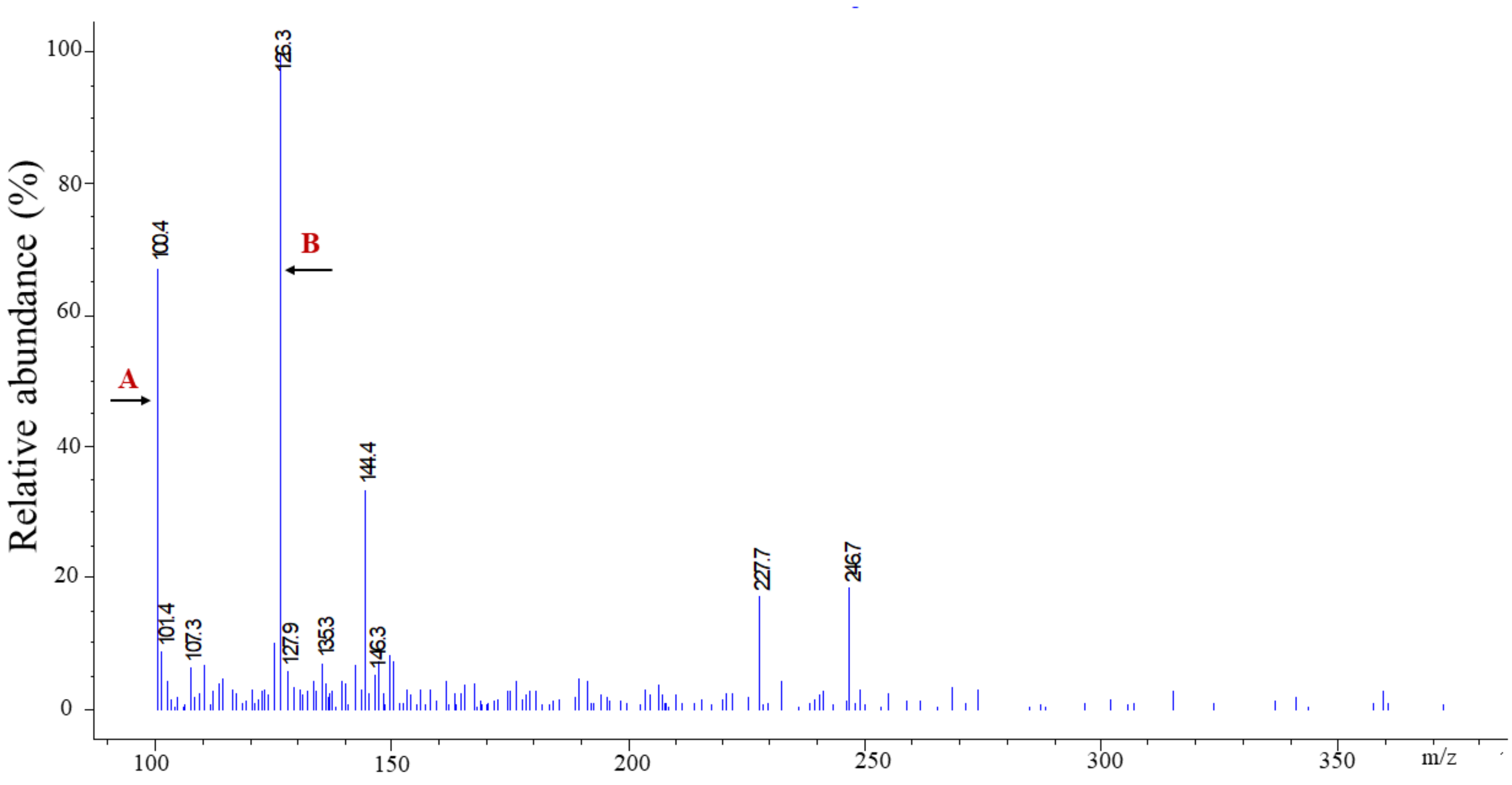

Fig. S13. Sample FABC2-1, t_R=53.136 min.

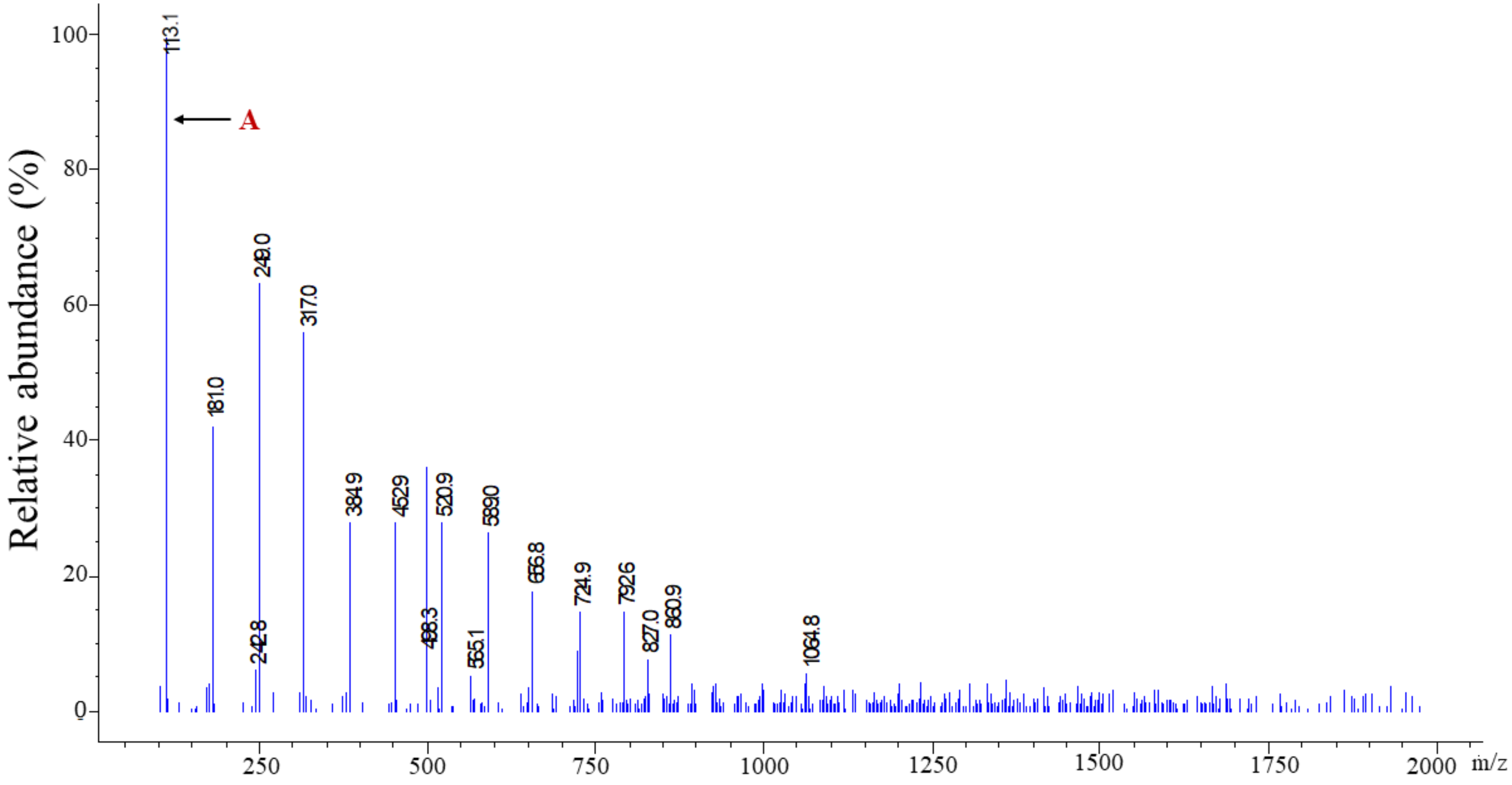

Fig. S14. Sample FABC2-2, t_R=5.794 min.

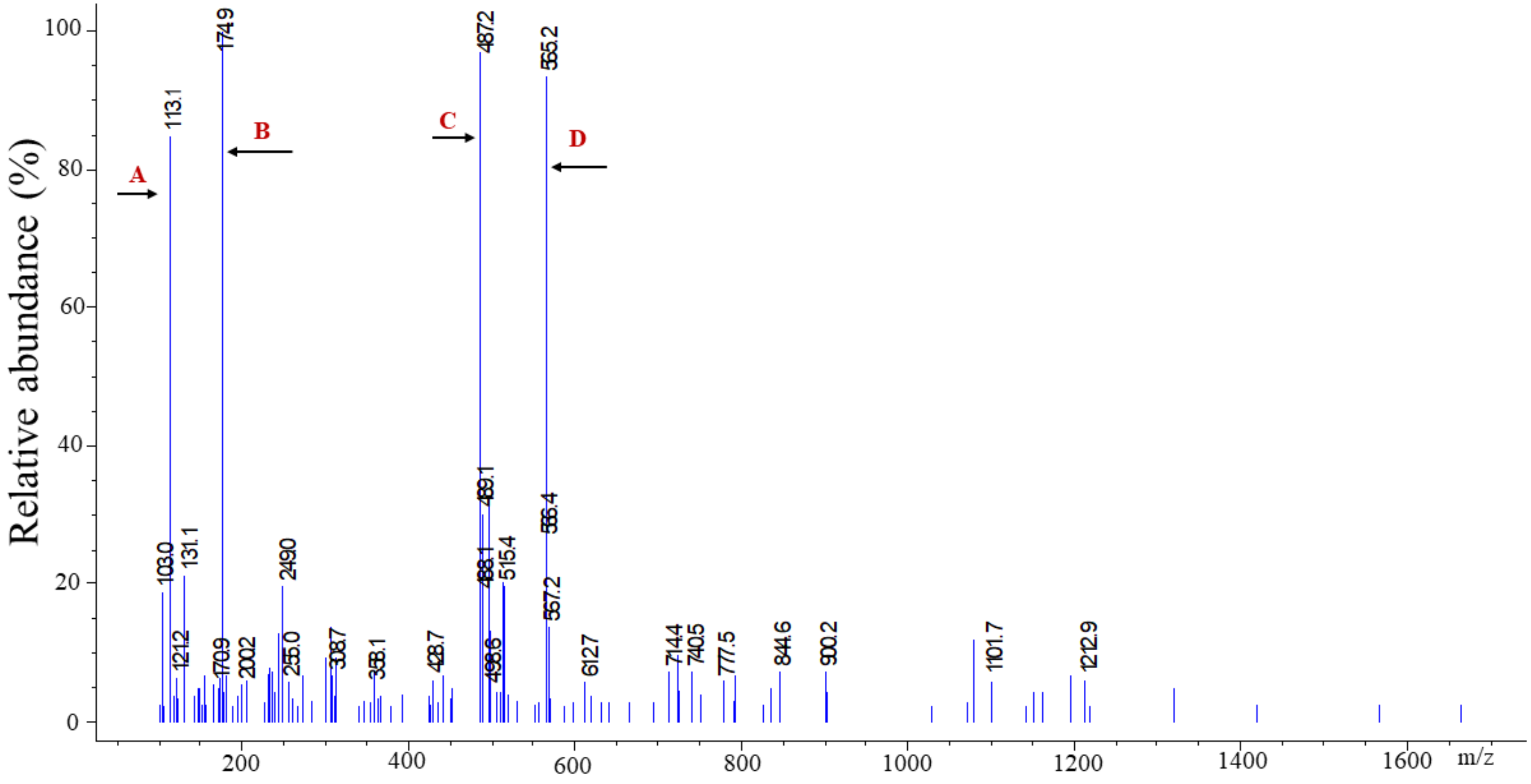

Fig. S15. Sample FABC2-2, t_R=6.127 min.

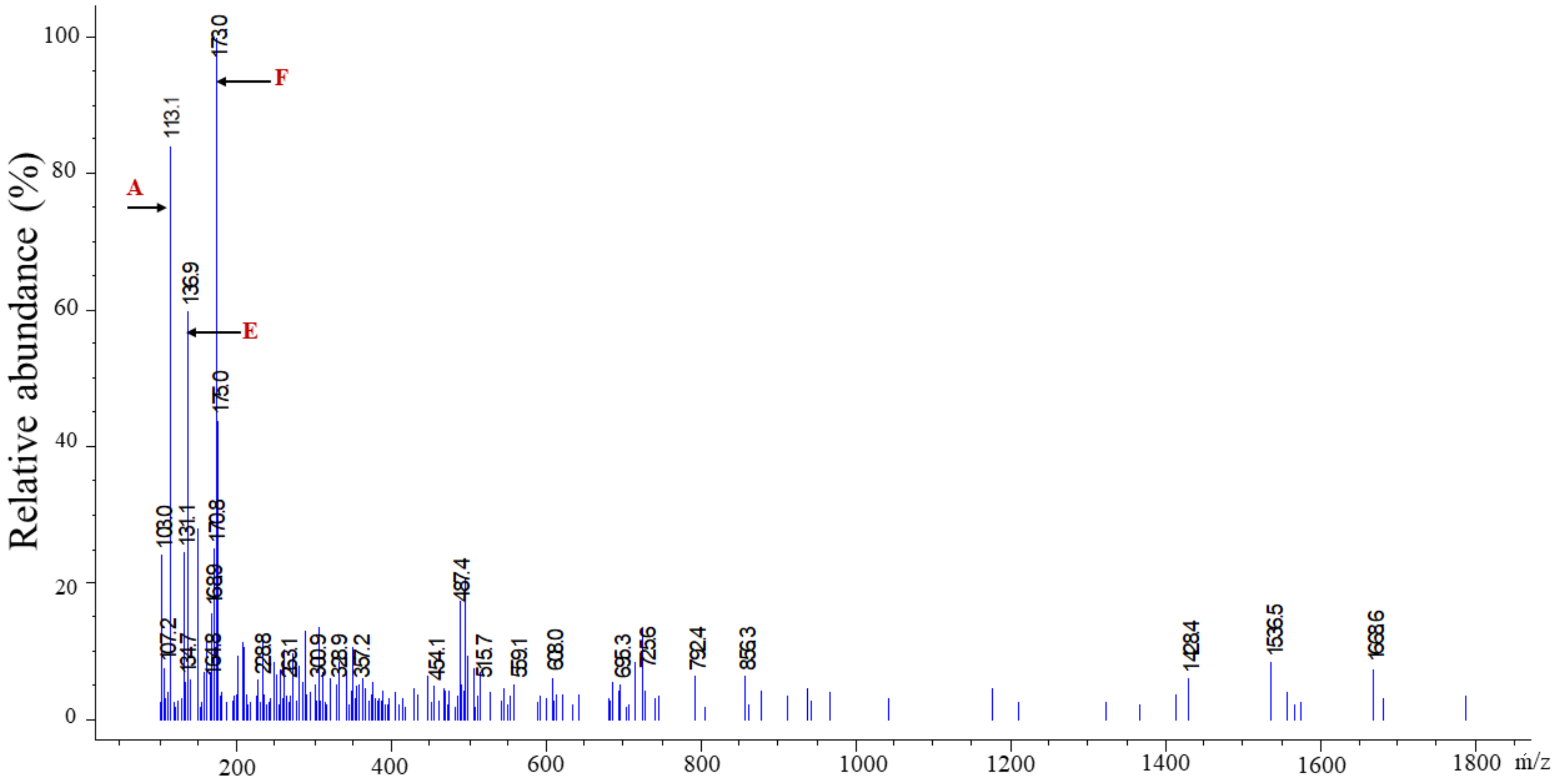

Fig. S16. Sample FABC2-2, t_R=9.148 min.

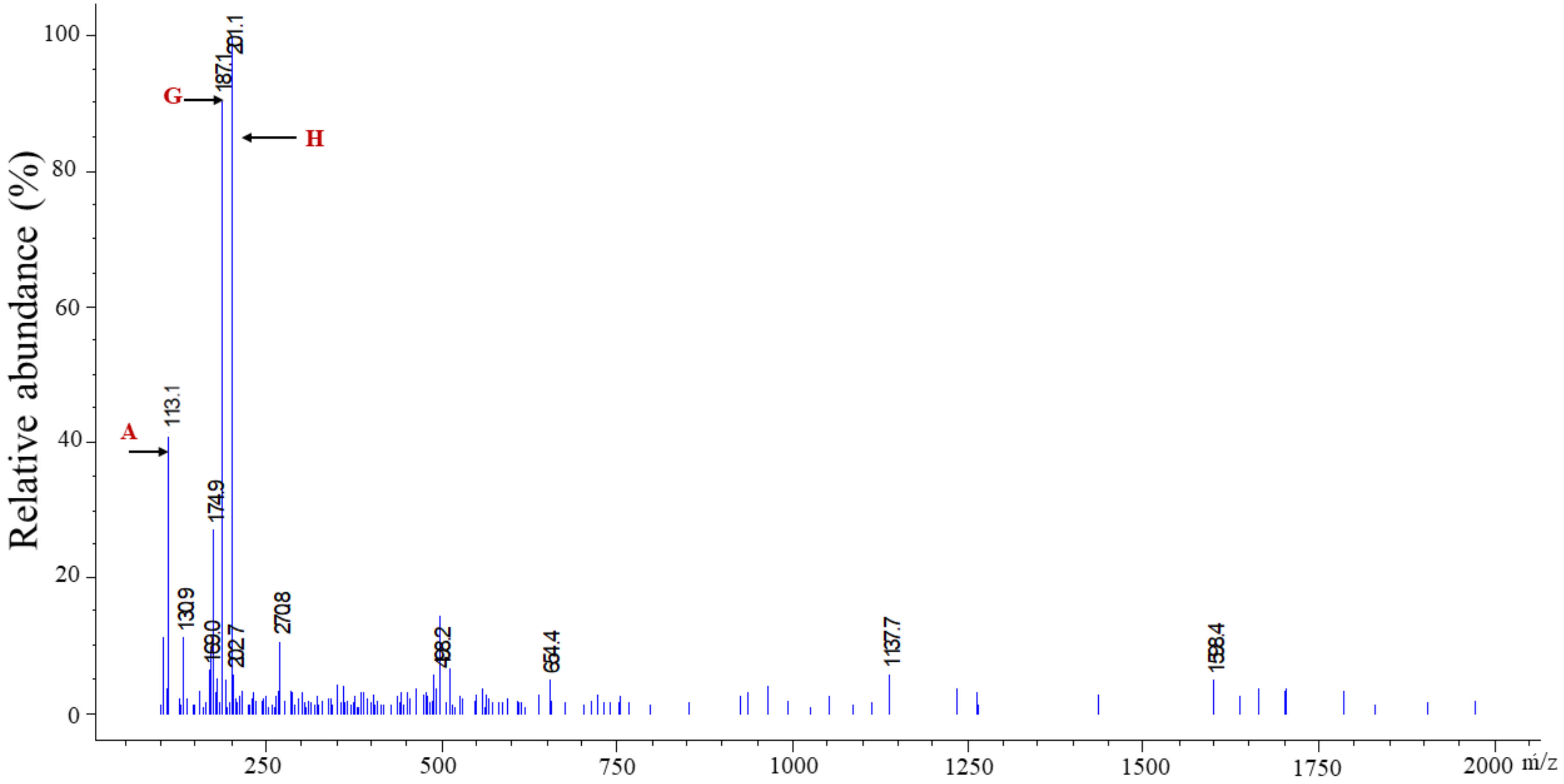

Fig. S17. Sample FABC2-2, t_R=10.095 min.

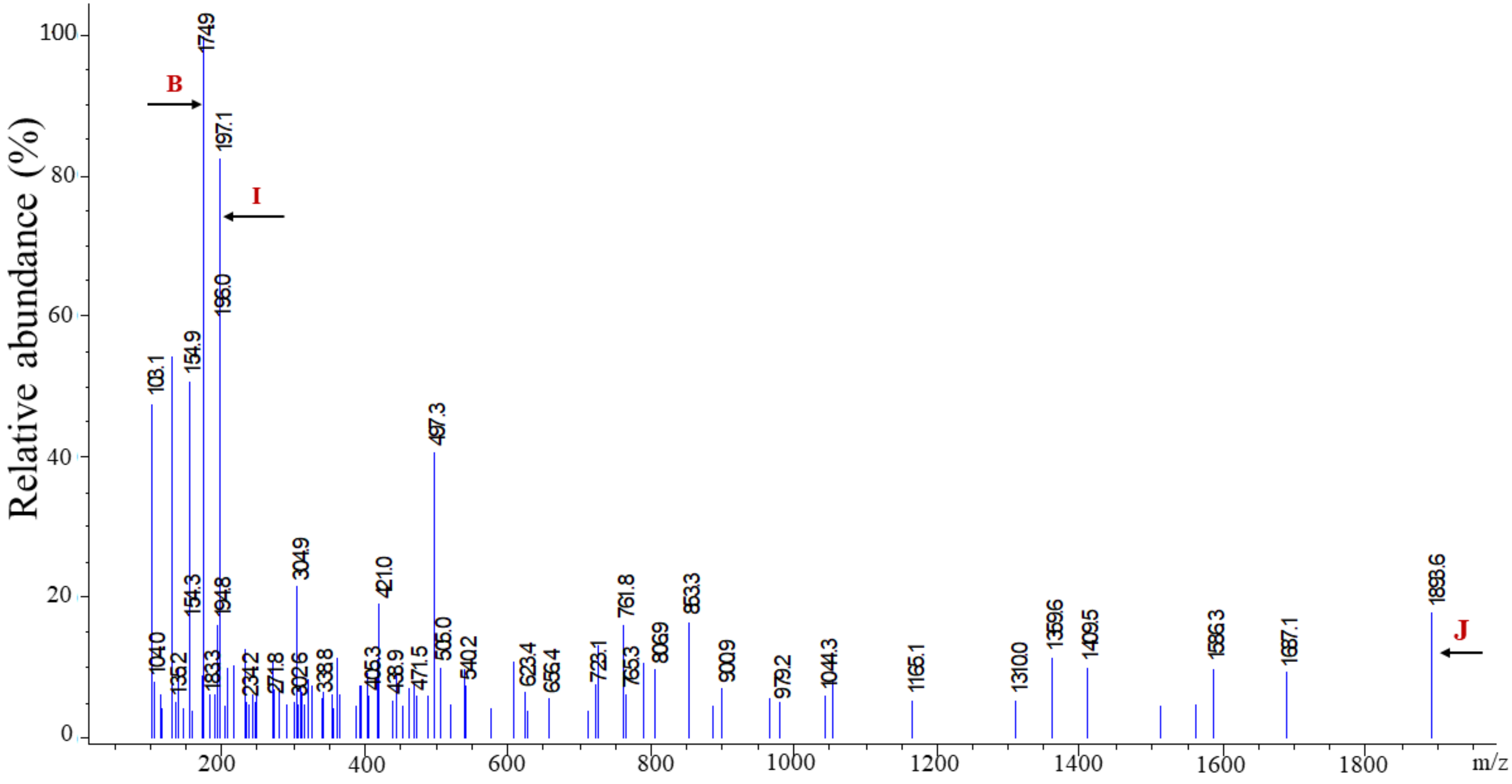

Fig. S18. Sample FABC2-2, t_R=15.995 min.

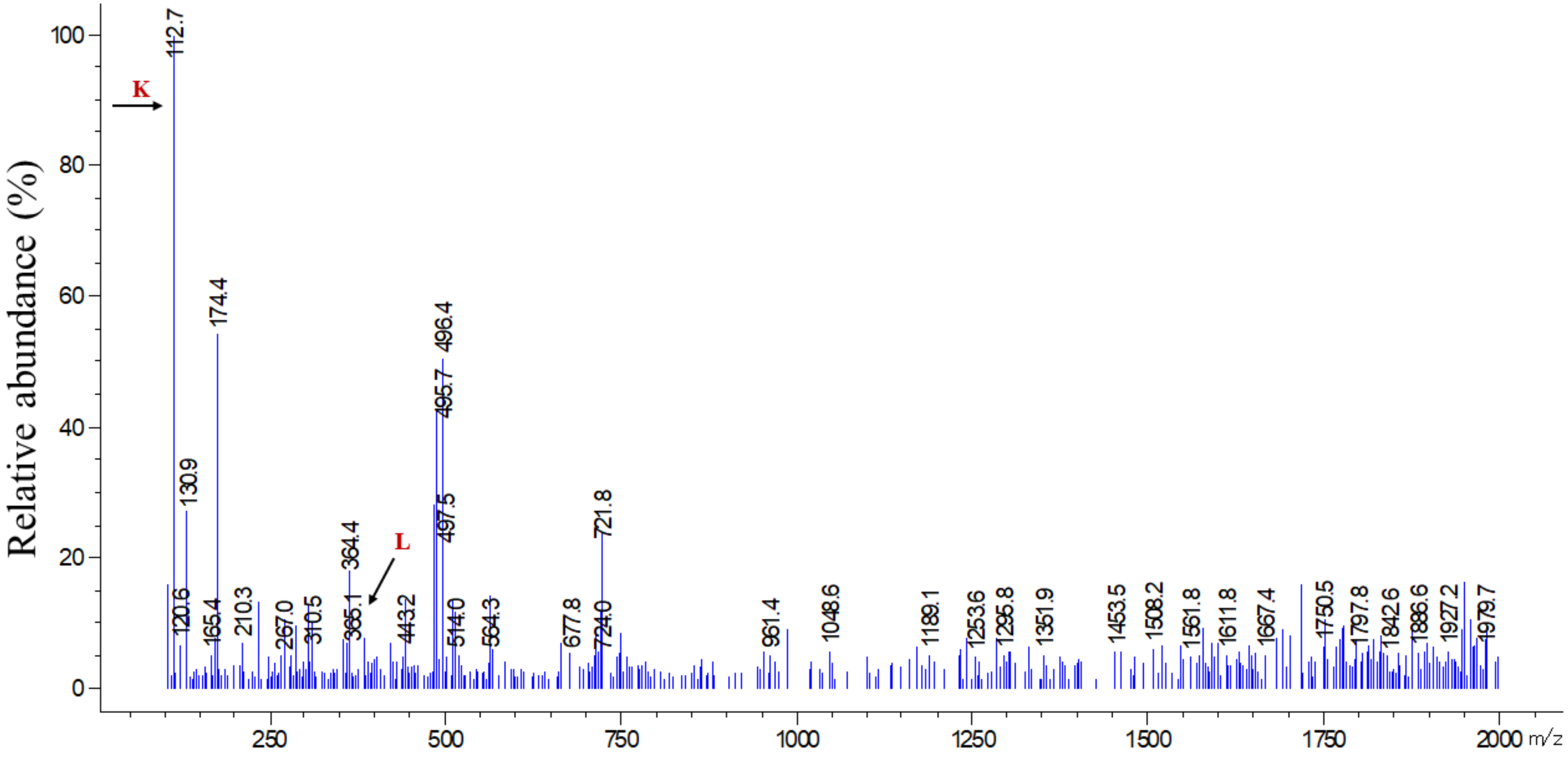

Fig. S19. Sample FABC2-2, t_R=29.053 min.

Table S5. Peaks from A to E obtained from the mass spectra in SCAN mode (Fig. S8 to S19) using ESI+ mode. The m/z ratios of A to E, their respective retention times (t_R) and the percentage of relative abundance of each peak are shown.

	ESI+	
Peaks	m/z	t_R (relative abundance (%) obtained from mass spectra)
A	100.4	5.025 min (100%), 9.208 min (100%), 13.492 min (39%) and 53.136 min (67.5%).
B	126.3	5.025 min (76%), 9.208 min (90%), 13.492 min (48%) and 53.136 min (100%).
C	123.2	6.073 min (100%).
D	475.3	13.492 min (100%).
E	149.0	14.965 min (100%).

Table S6. Peaks from A to L obtained from the mass spectra in SCAN mode (Fig. S24 to S29) using ESI- mode. The m/z ratios of A to L, their respective retention times (t_R) and the percentage of relative abundance of each peak are shown.

	ESI-	
Peaks	m/z	t_R (relative abundance (%) obtained from mass spectra)
A	113.1	5.794 min (100%), 6.127 min (85%), 9.148 min (84%) and 10.095 min (41%).
B	174.9	6.127 min (100%) and 15.995 min (100%).
C	487.2	6.127 min (97%).
D	565.2	6.127 min (94%).
E	136.9	9.148 min (60%).
F	173.0	9.148 min (100%).
G	187.1	10.095 min (90%).
H	201.1	10.095 min (100%).
I	197.1	15.995 min (82%).
J	1893.6	15.995 min (17.5 %).
K	112.7	29.053 min (100%).
L	365.1	29.053 min (7%).
